# Sex differences in cancer and immunotherapy outcomes: the role of androgen receptor

**DOI:** 10.3389/fimmu.2024.1416941

**Published:** 2024-05-28

**Authors:** Junzhe Zhao, Qian Wang, Alexandra F. Tan, Celestine Jia Ling Loh, Han Chong Toh

**Affiliations:** ^1^Duke-NUS Medical School, Singapore, Singapore; ^2^Division of Medical Oncology, National Cancer Centre Singapore, Singapore, Singapore; ^3^Department of Medical Oncology Cancer Hospital of China Medical University/Liaoning Cancer Hospital & Institute, Shenyang, Liaoning, China; ^4^Imperial College School of Medicine, London, United Kingdom; ^5^Sengkang General Hospital, Singapore, Singapore

**Keywords:** sex, immunotherapy, androgen receptor, tumour microenvironment, gender oncology

## Abstract

Across the wide range of clinical conditions, there exists a sex imbalance where biological females are more prone to autoimmune diseases and males to some cancers. These discrepancies are the combinatory consequence of lifestyle and environmental factors such as smoking, alcohol consumption, obesity, and oncogenic viruses, as well as other intrinsic biological traits including sex chromosomes and sex hormones. While the emergence of immuno-oncology (I/O) has revolutionised cancer care, the efficacy across multiple cancers may be limited because of a complex, dynamic interplay between the tumour and its microenvironment (TME). Indeed, sex and gender can also influence the varying effectiveness of I/O. Androgen receptor (AR) plays an important role in tumorigenesis and in shaping the TME. Here, we lay out the epidemiological context of sex disparity in cancer and then review the current literature on how AR signalling contributes to such observation via altered tumour development and immunology. We offer insights into AR-mediated immunosuppressive mechanisms, with the hope of translating preclinical and clinical evidence in gender oncology into improved outcomes in personalised, I/O-based cancer care.

## 1 Introduction

Differences in biological and sociocultural patterns between males and females have led to notable contrast in the characteristics of cancer pathophysiology. Research has revealed sex disparities in cancer incidence and prognosis, which are influenced by sex chromosomes and sex hormones, as well as distinct lifestyles, dietary habits, and environmental exposures (1). Since 2014, the National Institutes of Health have urged scientists to incorporate sex as a biological variable in their study design, aiming to reduce sex-related research biases (2). We now know that sex hormones play a crucial role in the initiation, progression, and treatment outcomes of cancer. Extensive studies are available on the crucial role of oestrogen and its pathways in the onset and progression of tumours, sometimes notwithstanding the oestrogen receptor (ER) status (3). On the other hand, the role of androgens and their signalling pathways on different cancers is less understood, except in prostate cancer. Emerging evidence on how androgen receptor (AR) affects tumour immunology has once again emphasised the significance of sex difference in response to antitumor therapies (4), prompting further investigation into this intriguing area.

This review collates current knowledge of the connection between biological sex and cancer epidemiology, the interplay between environmental and hormonal factors, AR and cancer sexual dimorphism, as well as the effect of AR on cancer immunology, before suggesting how AR contributes to immunotherapy resistance. Nevertheless, it is necessary to remain cognisant of how human *genders* - sociocultural constructs of the characteristics of men and women - exert significant influence on the lifestyles and exposures experienced by the two *biological sexes*, together shaping the apparent differences in immunotherapy response between males and females.

## 2 Epidemiology

**Figure 1:**
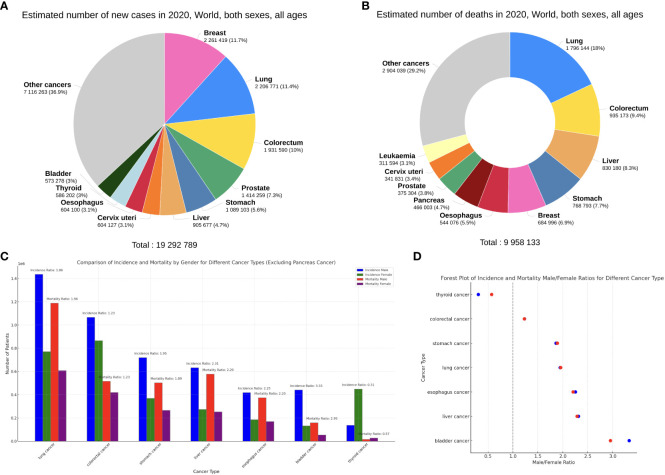
**(A, B)** Top 10 most common cancers worldwide (all ages and sexes) by incidence **(A)** and mortality **(B)**, plotted on the Global Cancer Observatory (GCO) platform (14). **(C)** The incidence and mortality of the 7 sex-neutral cancers in the top 10, females versus males. **(D)** Male/Female ratios of incidence (blue) and mortality (red) of the 7 cancers. Data from GLOBOCAN2020.

Recent studies have shown that females tend to have more potent immune functions than males (5), and their overly robust immune system can paradoxically be a double-edged sword that leads to increased occurrence of immune dysregulation (6–8). Therefore, sex has always been an important risk factor for certain infections (6), autoimmune disorders (9), cardiovascular diseases (10) and so on. However, whether certain cancers affect more males than females (or vice versa) remains a contentious topic (11, 12). Based on the GLOBOCAN2020 database (13) regarding the top 10 cancers by incidence and mortality (**Figures 1A, B**), we can observe that besides the more sex-specific cancers (breast, cervix, prostate), there are 6 male-dominant cancers (bladder, colorectal, liver, lung, oesophagus, stomach) and 1 female-dominant cancer (thyroid) (**Figures 1C, D**).

**Figure 2:**
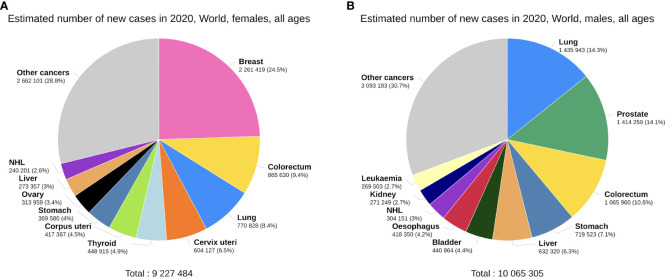
Top 10 most common cancers worldwide by incidence, females **(A)** versus males **(B)**, plotted on the GCO platform (14). Data from GLOBOCAN 2020.

Expectably, breast cancer occupies the foremost position in the incidence of cancers in females, accounting for 24.5% of new cancer cases, far more than colorectal cancer (CRC) at 9.4% (**Figure 2A**). Thyroid cancer (TC) is the only non-reproductive-related cancer that is female-dominant, with a male/female incidence ratio of 0.31 (**Figure 1D**). Importantly, however, when males do get TC, the male sex seems to be an independent negative indicator of TC prognosis. Data from Canada reveals that men with well-differentiated TC have a higher risk of recurrence than women, with a hazard ratio (HR) of 2.72 (15).

Compared with females, many of the common cancers occur more frequently in men (**Figure 2B**). Bladder cancer exhibits a notable sex disparity in incidence and mortality (**Figure 1D**), while females with non-muscle invasive bladder cancer have a higher risk of recurrence than males (16). This could potentially explain why the male/female mortality ratio is lower than the incidence ratio in bladder cancer. Liver cancer is another male-dominant cancer, ranking third in mortality globally (17). With a male/female incidence and mortality ratio of 2.31 and 2.29 (**Figure 1D**), the sex disparity is even more pronounced in East Asia (18). Other gastrointestinal tumours, including gastric, oesophageal, and colorectal cancers, also show higher incidence and mortality rates in males, consistent with the trends reported in literature (19–21). Lung cancer is also a male-dominant cancer; yet sex difference in lung cancer incidence is more pronounced within individual subtypes, with a greater male predominance in squamous cell carcinoma (17) and a notable East Asian female predominance in EGFR-mutated adenocarcinoma, the mechanisms of which are still not well understood (22). Notably, recent studies have reported a reversal of the sex disparity in lung cancer, where its incidence has become higher amongst young and middle-aged females (23) with more estimated new cases (17).

Notable sex disparities also exist in cancers with lower incidence rates. For instance, nasopharyngeal carcinoma (NPC) has a strong male predominance amongst Asian cancers, where the male/female incidence ratio ranges from 2:1 to 3:1 (24). Sexual dimorphism also exists in melanoma biology (3), with a male/female incidence ratio in melanoma ranging from 2:1 to 3:1 as well (25). Melanoma in males tends to be more aggressive, while female patients show better prognosis and longer survival (26–28).

## 3 Non-AR-related factors contributing to sex disparities in cancer incidence

### 3.1 Modifiable factors

As demonstrated earlier, males generally have higher incidence and mortality rates than females for bladder, colorectal, liver, lung, oesophagus, and stomach cancers (29). These sex disparities cannot solely be explained by the biological sex; lifestyle and environmental exposures are indispensable as well. In the UK, excluding sex-specific cancer types, modifiable risk factors account for 36.4% of male cancer cases and 25.6% of female cases. Tobacco smoking alone contributed to 15% of preventable cancer cases in the UK in 2015 and represents the highest proportion of preventable cancer cases in the US and Australia (30). Male and female smokers are 23 and 13 times more likely to develop lung cancer compared to non-smokers, respectively (31). Chronic alcohol consumption is also strongly linked to various cancers, with dose-response relationships seen in multiple epidemiological studies for liver, colorectal and upper aerodigestive tract cancers (32–34). Subgroup analyses in people with alcohol use disorders have shown that females have a higher risk of developing cancers compared to men (OR=1.767) (35). Additionally, consuming the same amount of alcohol leads to a greater increase in absolute lifetime cancer risk for women (1.4%) compared to men (1%), although these higher cancer rates in women may be attributed to breast cancer (36).

Obesity represents a major public health challenge, with approximately 55% of cancers in females and 24% in males in the USA considered obesity related. Importantly, 42% of new cases of overweight and obesity related cancers are gynaecological and breast cancers. This implies a stronger correlation between high body-mass index (BMI) and female cancers, highlighting the role of aromatase and oestrogen in gynaecological and breast cancer development (37). Non-sex specific cancers have a higher incidence in males, particularly oesophageal (male to female ratio of adenocarcinoma 4.4, squamous cell carcinoma 2.7) and colorectal cancers (38, 39). Obesity also plays a role in the tumorigenesis of these cancers, possibly involving chronic inflammation and systemic insulin and adipokine dysregulation (40, 41) that raise the incidence of metabolic syndrome (including metabolic dysfunction-associated steatotic liver disease, MASLD) particularly in males (42).

Globally, oncogenic viruses contribute to approximately 10% of all malignancies, although this varies between higher and lower income countries (43). The population attributable fractions are higher in females than males, primarily due to the inclusion of sex-specific cancers. Causative agents include human papillomaviruses (HPV), hepatitis B/C viruses (HBV/HCV), Epstein-Barr virus (EBV), and human immunodeficiency virus (HIV) (30). HPV^+^ head and neck squamous cell carcinoma (HNSCC) (44), EBV-driven NPC (24), as well as HBV/HCV-driven hepatocellular carcinoma (HCC) (45) all show a strong male predominance.

### 3.2 Sex chromosomes

Sex chromosome differences may also contribute to variations in cancer incidence. Females have XX and males have XY sex chromosome combinations, while intersex individuals such as those with Turner’s or Klinefelter’s syndrome have chromosomal patterns deviating from the typical configurations. In XX individuals, some pseudoautosomal genes can escape X-chromosome inactivation (XCI) providing a “buffering” effect against allele mutations. Incomplete XCI occurs in 23% of X chromosome genes (46). Thus, a single allele mutation leads to complete alteration of gene function in males, as opposed to a heterozygous alteration in females. This serves as a safeguard, preserving tissue function in the presence of mutations. Many of these genes, including *ATRX*, *KDM5C, KDM6A*, and *MAGEC3*, have tumour suppressor functions. Additionally, mutated alleles on the inactive X chromosome are typically expressed at lower levels or not expressed at all, mitigating their impact on cellular function (47). In females, the selective proliferation of specific mosaic subpopulations exhibiting preferential expression of one X chromosome can lead to skewed XCI (48). This can confer advantageous immunomodulation against cancer – a protective mechanism not available to males who obligatorily express the same mutated maternal X-linked gene.

X-linked genes, including *HUWE1*, *FLNA* and *MED12*, can directly modulate *TP53* expression. This association may render males at a higher risk of p53 dysfunction. Females exhibit a higher incidence of non-expressed mutations among p53-associated X-linked genes. Bioinformatic analyses in 12 non-reproductive cancers have shown that in females, less than half of these exome mutations were transcribed into mRNA, whereas the majority underwent mRNA transcription in males (49). These findings suggest tumour suppressor effects of the X chromosome.

Loss of Y chromosome (LOY) has been implicated in the pathogenesis of lung cancer, renal tumours and up to 40% of bladder cancer (50–52). In muscle invasive bladder cancer, patients exhibiting low Y chromosome gene expression of *KDM5D*, *KDM6C*, *TBL1Y* and *ZFY* demonstrate worse prognosis (52). Mosaic LOY in peripheral leukocytes is also associated with solid tumour incidence. Extreme downregulation of Y is linked to increased cancer risk and resistance against EGFR tyrosine kinase inhibitors (53), which may also impact immunotherapy response downstream. Loss of the entire X chromosome(s) has been documented in early-stage astrocytoma, neuroblastoma and medulloblastoma (54–56).

### 3.3 Oestrogen and ER

The link between oestrogen or ER and non-reproductive cancers is unclear. At the molecular level, oestrogen and ER affect PD-1 signalling, Wnt/β-catenin pathways and the Ras/MAPK pathway, amongst many other aspects of cancer biology (57–62).

Circulating E1 (oestrone) and E2 (oestradiol) levels were found to have no statistically significant relationship with colon cancer in a cohort of 1000 postmenopausal women (62). However, a 2015 meta-analysis revealed a reduced ratio of ERβ expression in CRC compared to the normal mucosa (OR=0.216), associated with poorer overall and disease-free survival (63). Conversely, exogenous oestrogen reduces the risk of CRC by 37% as demonstrated by the landmark Women’s Health Initiative study (64). *In vitro*, ERβ was shown to modify the hypoxic response by downregulating HIF-1α, VEGFA and PDGF (65).

Oestrogen plays a complex role in the liver. It has been implicated in various liver pathologies like fibrosis and fatty liver disease, but its role in HCC remains unclear. In a cohort of 275 men, higher total E2 is associated with increased HCC risk (OR=1.58) (66). A recent cohort study shows a survival advantage for female HCC patients over males in perimenopausal and early-menopausal ages but not in postmenopausal women, possibly due to declining endogenous oestrogen production (67). However, female patients in phase III trials for immune checkpoint inhibitors (ICI) for HCC are found to have worse overall survival (OS) than males (68). Whether this discrepancy can be attributed to oestrogen is unclear. Studies exploring the use of tamoxifen in HCC have yielded mixed results, with some showing prolonged survival but larger studies finding no significant association (69–71).

In lung cancer, oestrogen appears to have a protective effect. A meta-analysis of female lung cancer cases demonstrates that higher levels of sex steroid hormone exposure, both endogenous and exogenous, reduce lung cancer risk by 10% (72), yet the role of ERα or ERβ is unclear. Some studies suggest that ERα is associated with worse prognosis in non-small cell lung cancer (NSCLC) (73), while others find no significant effect. Some meta-analyses indicate an association between ERβ and better prognosis in NSCLC (73, 74), while others consider it an unreliable prognostic marker (75, 76) depending on the methods employed, such as uni- vs multivariate analysis, bioinformatics, or immunohistochemistry (IHC) analysis. Finally, female reproductive factors like breastfeeding are associated with a decreased risk of oesophageal and gastric adenocarcinoma, though parity, menstruation, and the use of hormone replacement therapy have no association (77). Interestingly, the use of tamoxifen, a selective oestrogen receptor modulator (SERM), is associated with an increased risk of gastric adenocarcinoma (78) as well as endometrial cancer. The tissue-specific agonist/antagonist role of SERMs like tamoxifen reflects the complex role of the oestrogen-ER signalling axis in tumorigenesis.

## 4 Androgens, AR, and tumour pathophysiology

### 4.1 Androgens and non-reproductive cancers

Androgens include testosterone, dihydrotestosterone (DHT), and dehydroepiandrosterone (DHEA), among others. Testosterone produced by the testes plays a pivotal role in initiating the development of masculine traits, hence exists in higher levels in males and lower in females. Androgen deficiencies in males can result in the development of feminine traits (79), while increased androgen production in females can lead to a shift from feminine to masculine traits and also be associated with polycystic ovarian syndrome (PCOS) (80). Their biological functions are executed by binding with AR and activating intracellular AR signalling downstream. Besides prostate cancer, the role of androgens in tumorigenesis is less studied compared to oestrogen. Higher concentrations of testosterone are associated with increased risk of liver cancer, particularly in men, while higher levels of DHEA, the adrenal precursor, are associated with a 53% decrease in risk (66, 81). Higher circulating testosterone is associated with a decreased risk of CRC in men, but this is not shown in women (81). The association between testosterone and oesophageal cancer is unclear, with varying degrees of significance across studies (81, 82). Gastric, pancreatic and bladder cancers are also shown to have no significant association with testosterone levels (81). Interestingly, androgen deprivation therapy (ADT) using finasteride has shown improved survival in patients with non-muscle invasive bladder cancer, suggesting a potential strategy to reduce bladder cancer incidence and recurrence (83).

### 4.2 Overview of AR

**Figure 3:**
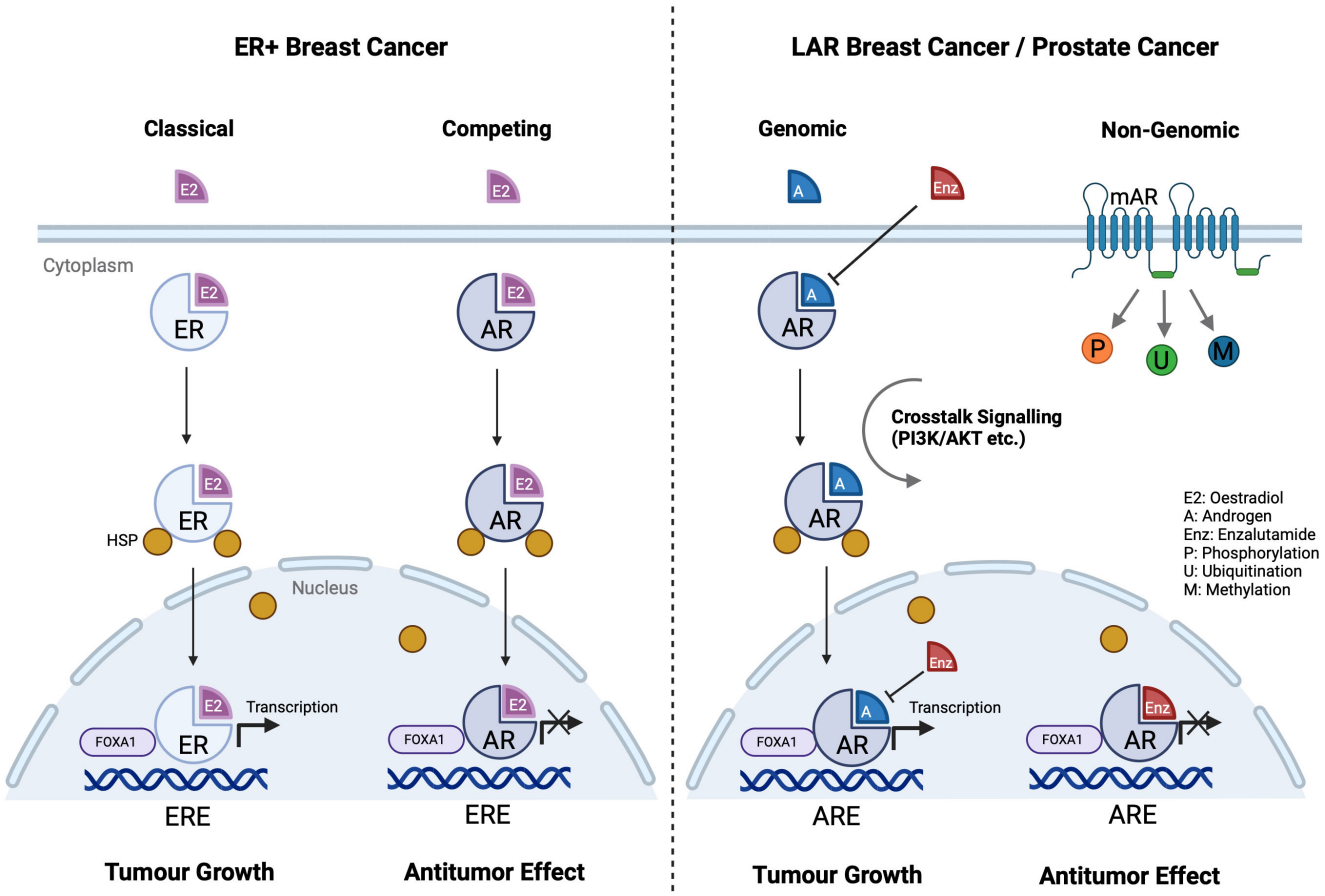
AR and ER signalling pathways in different cancer cells. ER and AR share similar structures, and they compete in each other’s signalling pathways. In ER+ breast cancer cells, AR substitutes ER on ERE and stops downstream transcription, eliciting an antitumor effect. ER and AR also share the same co-activator FOXA1 on ERE. In LAR breast cancer and prostate cancer, enzalutamide competes with androgen to stop AR activation. (Created with BioRender.com).

AR is a member of the nuclear receptor superfamily acting as a ligand-dependent transcription factor (84). Consisting of eight exons, the AR gene is located on the X chromosome. It comprises a ligand-binding domain (LBD), a DNA-binding domain (DBD), and an N-terminal domain (NTD). In the unbound state, AR forms a complex with co-chaperones, heat shock proteins, and cytoskeletal proteins in the cytoplasm. Ligand binding induces conformational changes, receptor dimerization, and translocation to the cell nucleus. The NTD influences transcriptional activity, while the DBD allows binding to and recognition of androgen response elements (AREs) on target genes where it serves to induce or repress gene expression through binding to chromatin at *cis* AREs (85). AR can also modulate post-translational modifications by phosphorylation, methylation, or ubiquitination (86, 87) (**Figure 3**). While AR exerts effects mostly in sex hormone-dependent tissues, such as the prostate, testes, ovaries, and endometrium (88, 89), it is also widely expressed in kidneys, liver, urinary bladder, as well as the cardiovascular, immune, musculoskeletal and nervous systems (88, 90–94). It is also noted that membrane androgen receptors (mARs), such as ZIP9 and GPRC6A, are a group of G protein-coupled receptors that directly alter cellular signalling upon androgen stimulation, also known as the non-genomic pathway (95, 96) (**Figure 3**). While studies have demonstrated the implications of mARs on prostate cancer, they are beyond the scope of this review.

A report of teenagers developing hepatocellular carcinoma due to excess androgen intake have spurred interest in the effect of androgen and AR on cancer (97, 98). In 1980, an article published in *The Lancet* highlighted the association between elevated levels of free testosterone in males and an increased risk of melanoma (99). While multiple observations support the hypothesis that excess androgens may be tumorigenic (100), a definitive mechanistic explanation is still lacking, which necessitates our summary of current knowledge below.

### 4.3 AR and tumour development/progression

AR signalling is the primary driver of castration resistant prostate cancer (CRPC) (101). Enzalutamide, an AR antagonist, competes with androgens to bind to AR and blocks nuclear ARE binding, thereby inhibiting downstream transcriptional activity (102) and enabling antitumor effect (103). AR and ER exhibit similarities as nuclear receptors, allowing substantial signalling crosstalk (**Figure 3**) (104). In ER+ breast cancer, AR competes with ER for oestrogen response elements (EREs) and inhibits ER activity, playing a tumour-suppressive role especially in premenopausal patients (105). However, AR may promote cancer progression in certain ER– breast cancers. A study indicated that the luminal AR (LAR) subtype accounts for 15% of triple-negative breast cancer and AR is an attractive therapeutic target (106). Higher AR expression and corresponding aggressive phenotypes are observed predominantly in tissue samples from African American women, with a strong interaction between AR and JAK-STAT signalling (107). Another study shows that *PIK3CA* is highly mutated in the LAR subtype, where PI3K inhibitors can reduce LAR cell proliferation (108). Salivary duct carcinoma (SDC), a male-dominant cancer, is a rare, aggressive malignancy also characterised by high AR expression, ranging from 70% to 97.8% (109–111). Recent studies have found that the occurrence of SDC is closely related to the AR signalling pathway (112), sharing similar molecular profiles with high-grade breast ductal carcinoma and apocrine breast cancer (113). AR-V7, an AR splicing variant, accounts for over 50% of AR in SDC and plays a crucial role in the resistance and progression in CRPC (114). Other studies have reported that *FOXA1* mutations are present in 10% of SDC cases, resulting in drug resistance and tumour progression also via the AR pathway (113).

With its homolog crucial for primary sex determination in *C. elegans* (115), FOXA1 is a key transcription factor necessary for AR and ER activities in prostate and breast cancers (116). AR driven transcription in molecular apocrine breast cancer is mediated by FOXA1 (117). In prostate cancer, *FOXA1* exhibits a high mutation rate, thereby affecting AR transcription (118). Elevated levels of FOXA1 have been associated with poor prognosis in prostate cancer. FOXA1 function in AR signalling and its impact on prostate cancer differs markedly from its role in ER signalling and breast cancer progression (119). A study published in 2012 highlights the significance of FOXA1 and FOXA2 in sexual dimorphism in liver cancer, noting that modulation of these factors can reverse the observed gender differences (120). Other *FOX* family genes are also crucial in regulating the PI3K-AKT-mTOR pathway. FOXO3a, a PI3K/AKT downstream substrate, can induce AR expression as a positive regulator (121). FOXO1, a downstream effector of AR, can also lead to AR hyperactivation in prostate cancer with PTEN loss, independent of androgen binding (122).

The crosstalk between AR and other signalling pathways has also been reported (123, 124). With a strong association between nuclear AR expression and Wnt/β-catenin signalling in bladder cancer, ADT has shown great therapeutic potential (124). Moreover, TCF1 and AR have overlapping binding sites on β-catenin (125). β-catenin translocates into the nucleus and interacts with TCF1 and lymphoid enhancer factor, activating the transcription of target genes. TCF1 is required for the self-renewal of stem-like CD8+ T cells in response to viral or tumour antigens, preserving heightened responses to checkpoint blockade immunotherapy (126). This implies not only a causal relationship between AR signalling and tumour progression via β-catenin pathways, but also a connection between AR and antitumor immune responses (more in Section 5.2). In addition, androgens can also influence the effectiveness of BRAF-targeted therapy in melanoma. AR expression is elevated in BRAF-resistant melanoma. Inhibition of both the AR and BRAF/MEK pathways counteracts resistance and hence improves cytotoxicity (127). Intriguingly, blocking AR not only inhibits the proliferation of BRAF-resistant cells, but also enhances the infiltration of CD8+ T cells and promotes cancer cell apoptosis (128). This prompts further investigation on how AR affects immune responses, and targeting AR may offer new combination therapies for cancer treatment.

## 5 AR and cancer immunotherapy

### 5.1 Sex difference in clinical trial outcomes

There are several meta-analyses evaluating the comparative efficacy of immuno-oncology (I/O) on various cancers across genders (**Table 1**). A 2018 meta-analysis summarises 20 clinical trials involving ICIs across various cancer types, with a total of 11,351 participants (129). These trials predominantly focus on melanoma (32%) and NSCLC (31%). The meta-analysis reveals significant sex differences in clinical outcomes, where females experience lower response rates than males. However, the significant heterogeneity calls for analysis specific to individual cancer types and treatments. In 2019, the same team conducted another meta-analysis of chemotherapy and I/O for advanced lung cancer; this time with opposite conclusions compared to a year ago (130). Women with advanced lung cancer seem to derive a larger benefit from the addition of chemotherapy to anti-PD-1/PD-L1 compared with men. Another meta-analysis on NSCLC patients receiving combination chemo-immunotherapy first-line also concludes that females show a more significant improvement in OS and progression-free survival (PFS) (132). These findings highlight the potential impact of gender on the effectiveness of both targeted and combination of chemo-immunotherapies in NSCLC.

**Table 1:** Summary of meta-analyses evaluating the efficacy of I/O interventions across males and females on different cancers.

Cancer Type	# Patients	Year	Reference	Comparative Arms	Summary of Findings
Advanced solid tumours • 32% melanoma • 31% NSCLC	11,351	2018	Conforti et al (129)	ICI compared with others	Females show lower response rates
Advanced NSCLC/SCLC Advanced NSCLC	4,923 3,974	2019	Conforti et al (130)	ICI + chemotherapy VS chemotherapy ICI alone OR ICI + chemotherapy	Females benefit more from the ICI + chemotherapy combination
Advanced solid tumours	10,664	2020	Wei et al (131)	13 ICI-alone regimens 5 ICI-based combinations	EGFR mutations are more likely to occur in females No sex difference for ICI monotherapy benefits Females benefit more from ICI-based combinations
Advanced/recurrent NSCLC	5,830	2022	Takada et al (132)	ICI + non-ICI VS non-ICI	Females show greater benefit in OS and PFS when receiving combined chemoimmunotherapy
Advanced HCC	5,169	2023	Balcar et al (68)	ICI alone OR ICI-based combinations	Females show smaller (pooled) OS benefit from ICI- based therapy Comparable outcomes for Atezo/Bev (on a real-world cohort of 840 patients)
mRCC mUC	4,206 (mRCC) 2,240 (mUC)	2023	Yanagisawa et al (133)	ICI-based combination VS TKI ICI-based combination VS chemotherapy	PFS and OS benefit seen in first-line ICI-based combination; no difference between the sexes OS benefit seen in first-line ICI-based combination; no difference between the sexes

mRCC, metastatic renal cell carcinoma; mUC, metastatic urothelial carcinoma.

In 2020, a meta-analysis on NSCLC includes 13 studies with monotherapy and 5 with combination regimens (KEYNOTE 010/024 with pembrolizumab versus chemotherapy and CHECKMATE 017/026/057 with nivolumab versus chemotherapy), a total of 1028 female and 1435 male patients (131). The result confirms that EGFR wild-type patients could benefit from immunotherapy monotherapy (HR=0.77; *p*<0.001) while those of mutant types experienced no survival benefit (HR=1.11; *p*=0.54). While EGFR mutations are more likely to occur in females (134), there is no apparent efficacy-sex association overall (131). Therefore, to explore the effect of AR in I/O efficacy, confounding factors such as mutations will need to be properly controlled and stratified.

There are no sex differences in the superior OS benefits from first-line ICI-based combination therapies in metastatic renal cell carcinoma (RCC) or metastatic urothelial carcinoma (UC) (133). In locally advanced RCC, however, adjuvant I/O monotherapy reduces recurrence risk in female patients (HR=0.71, 95% CI 0.55–0.93) but not in male patients (133). On the other hand, males with muscle-invasive bladder cancer have better DFS on adjuvant I/O compared to females (133). A meta-analysis on HCC shows single-agent I/O exhibits less OS benefit in females than males. On the other hand, combination atezolizumab-bevacizumab (Atezo/Bev) – a first-line standard of care for advanced HCC – yields comparable efficacy between males and females in a real-world cohort (68). Nevertheless, these studies did not conduct stratified analysis based on AR expression, which may have overlooked the importance of AR in sex disparity.

Other phase III trials in advanced urothelial, hepato-pancreato-biliary and upper or lower gastrointestinal tract cancers have also been individually screened for outcome differences in patients treated with ICI based on sex or AR levels (135–145). However, none of these trials made explicit analysis on how sex or AR affects the outcomes in these cancers. Importantly, though, one study on 23,296 patients enrolled in SWOG trials shows a 49% increased risk of adverse events (AE) in females receiving I/O, especially of haematological AEs (146, 147). It is hoped that more prospective studies on the relationship between sex, AR expression and I/O efficacy and AEs can be carried out to further explore the role of AR signalling in cancer immunology and immunotherapy.

### 5.2 AR and cancer immunology

**Figure 4:**
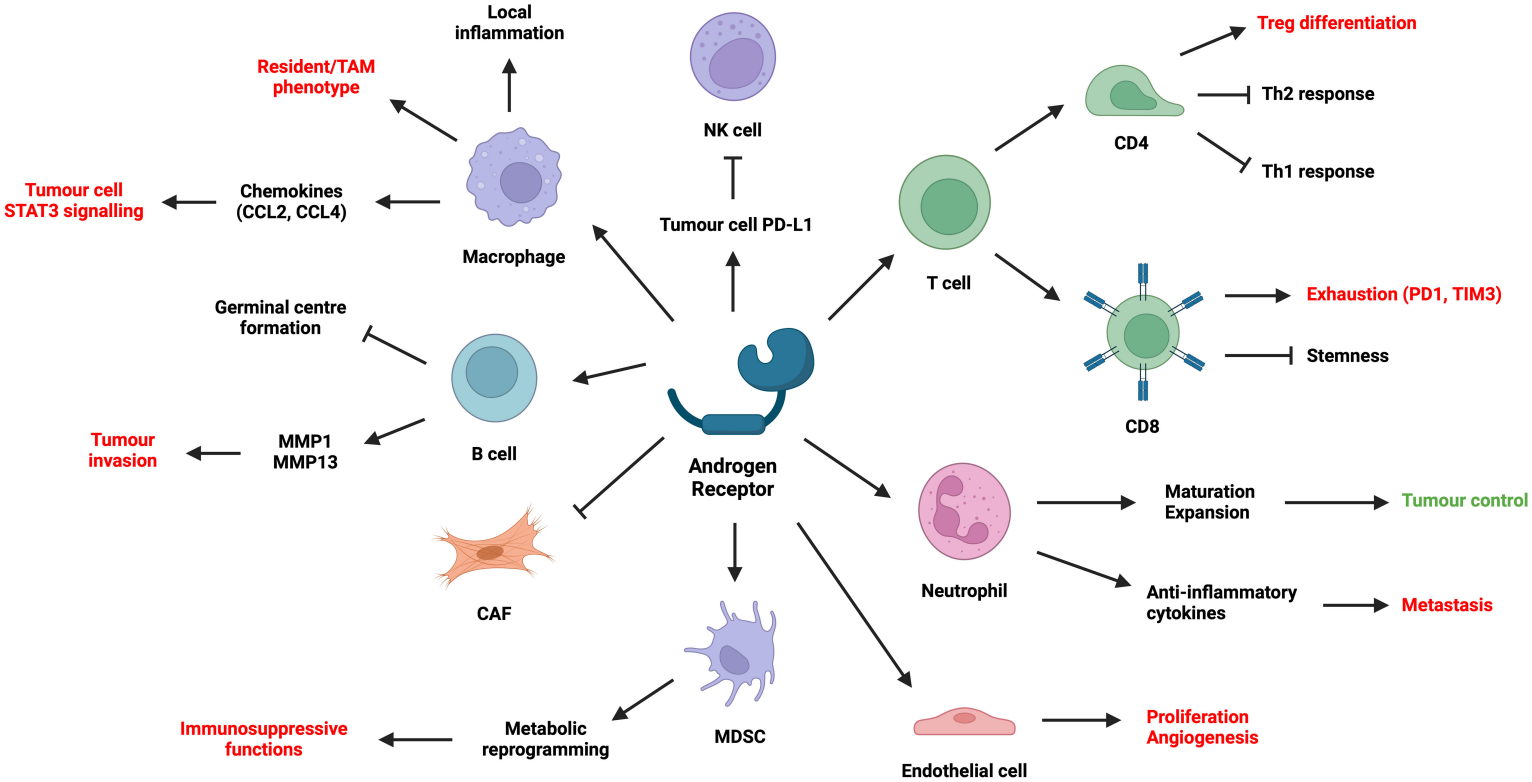
Summary of the effect of AR on different cell types in the TME. (Created with BioRender.com).

While previous sections have attempted to address how genetic, environmental, and hormonal effects lead to distinct tumorigenesis and disease progression patterns between males and females, studies over the past decade have emerged to explain how AR within the tumour microenvironment (TME) conspire to this process and alter patient response to treatments such as ICIs. This section summarises the effect of AR signalling in different TME cell types, laying the foundation for subsequent discussion on another dimension of I/O resistance (**Figure 4**).

Although AR signalling plays a key role in tumour immunosuppression, it is important to note the caveat when interpreting preclinical studies involving AR and biological sex. Cells harvested from male subjects have long been exposed to androgens and AR signalling. Hence, when manipulating AR-related pathways, male cells may behave very differently compared with female cells. Consequently, when designing and analysing clinical trials for I/O and antiandrogen combination, it is indispensable to include stratification based on biological sex, along with other variables such as circulating androgens, AR mutation/amplification/IHC status, and PD-L1 scoring.

#### 5.2.1 AR signalling in lymphocytes

In murine models of CRC and melanoma, male mice have more aggressive tumours which seemingly depend on CD8^+^ TILs (4, 148). AR signalling inhibits CD8 T cell stemness by regulating the epigenetic programme of T cell differentiation (4), while reducing IFNγ secretion via USP18 which inhibits NF-κB activation (148). This causes male TILs to be more terminally exhausted (PD1^+^TIM3^+^) with a loss of stem-cell like state (TCF1^-^). Surgical castration in combination with ICI improves tumour control. These results correlate well with CRC and melanoma patient data, where AR positively correlates with PD1 and TIM3 expression in CD8^+^ TILs. In addition, AR signalling transactivates *Tcf7*-centred regulons and directly results in the exhaustion of TCF1^+^ progenitor CD8^+^ T cells in murine bladder cancer (149). CD8^+^-specific *Ar*-KO or systemic use of enzalutamide reduces tumour burden, while combining castration with ICI improves tumour control. These processes do not seem to depend on sex chromosomes, but more on androgen exposure and AR signalling.

Evidence is clear that AR signalling in male CD4^+^ T cells suppresses Th1 and Th2 responses and favour T_regs_. AR signalling stabilises *Foxp3*^+^ T_regs_ during allergen challenge in males (150), possibly via a functional ARE within the *Foxp3* locus (151). Androgens also reduce the differentiation towards Th2 (150) and suppress Th2 functions in males (152), a consequence of AR binding to *Dusp2*. Androgen exposure also reduces Th1 differentiation by inhibiting IL-12 signalling (153). Pan-T cell *Ar*-KO renders severe airway inflammation in male mice during allergen exposure (150, 152).

One recent study has shown that while there are more NK cells in males, they often exhibit reduced cytotoxicity and tumour control (154). Such effect depends on both epigenetic factors (e.g. UTX) on the X chromosome (154) and the effect of sex hormones on tumour cell PD-L1 expression (155, 156). Specifically, high-dose androgen treatment on prostate cancer cells upregulates circFKBP5, which increases their PD-L1 expression and hence NK suppression (155). On the other hand, antiandrogens on bladder cancer cells reduce PD-L1 expression via ADAR2, which in turn increases NK cell cytotoxicity (156). Sorafenib treatment on HCC cell lines also enhances NK cell killing by reducing AR expression, leading to increased IL-12A secretion and NK activation. Further research is needed on the direct effects of AR signalling on NK cells.

There are limited findings on how AR signalling impacts B cell function. Androgens partially facilitate B cell migration away from the follicle centre via CCL21-GPR174 interaction, which prevents germinal centre formation (157). B cell proliferation and IgE synthesis are increased either by reducing circulating androgens (158) or by AR knockout (159), yet these effects do not enhance airway inflammation in allergen challenge (158). In another study, IL-8 increases AR expression on B cells, which promotes bladder cancer cell invasion by upregulating B cell expression of MMP1 and MMP13 (160).

#### 5.2.2 AR signalling in macrophages, DCs, and MDSCs

The direct role of AR signalling on myeloid cell phenotype and function remains a contentious area of research. Androgens upregulate TREM1-associated signalling pathways in THP-1 and induce resident-like phenotypes, promoting prostate cancer cell migration and proliferation (161). Enzalutamide reduces immunosuppressive tumour associated macrophages (TAMs) in prostate cancer patients (161). Nevertheless, while AR signalling in macrophages can increase prostate tumorigenesis via increased CCL4 (162) and consequent STAT3 activation, blocking AR in TAMs or prostate cancer cells may actually promote metastasis via CCL2/STAT3-mediated macrophage recruitment (163). Furthermore, in atherosclerosis (164) and wounds (165), AR signalling promotes local inflammation by enhancing TNFα expression, monocyte differentiation and chemotaxis (166), as well as foam cell formation via altered lipid metabolism. AR signalling in alveolar macrophages also promotes M2 macrophage-mediated eosinophilic inflammation, increasing lung damage in asthma mouse models (167). Hence, the role of AR signalling in macrophages depends not only on its direct effects, but also on the local tissue and disease contexts.

Though analysis has shown that ADT may lead to increased infiltration of myeloid-derived suppressor cells (MDSCs) into the TME (168, 169), there have been few studies looking at the direct effect of AR signalling on MDSCs or dendritic cells (DCs). B16 and 4T1 implantation results in higher tumour burden in female mice that is correlated with a higher plasmacytoid DC infiltration and less MDSCs compared with male mice (170). Functions of these tumour-associated DCs could depend on FOXO3-regulated AR/ER expression (170). In another seminal study, AR knockout or antagonism on MDSCs facilitate MC-38 tumour progression in mice, resulting from pAMPK-mediated metabolic reprogramming (171). Increased glycolysis and decreased mitochondrial respiration led to immunosuppressive MDSC phenotype (171), which has well been established (172).

#### 5.2.3 AR signalling in neutrophils

Research has shown that androgens promote neutrophil maturation and expansion in the bone marrow, as well as subsequent chemotaxis towards foci of injury or malignancy (173–175). AR-KO mice are often neutropenic and susceptible to acute bacterial infection (176). Male mice castrated prior to melanoma implantation also show impaired neutrophil maturation and function, with elevated metastatic burden that can be ameliorated by rescue testosterone replacement (174). Conversely, women with PCOS and insulin resistance often show increased circulating androgens associated with raised neutrophil count (177). Interestingly, in another study, ADT suppresses neutrophil cytotoxicity via increased TGFβ-RI (178), which is also seen in prostate cancer patients receiving ADT (174). High dose androgens or TGFβ-RI inhibition rescue AR-mediated neutrophil suppression and restore its anti-tumour effects (178).

However, androgen-sensitised neutrophils can also exhibit reduced bactericidal functions or cytotoxicity, hence promoting tumour progression. This phenotype is accompanied by high expression of anti-inflammatory cytokines such as IL-10 (175). For instance, AR signalling promotes hepatic neutrophil accumulation and contribute to MC-38 and B16 liver metastases (LM) (173). Antagonising neutrophil AR signalling axis significantly mitigates LM. Two other studies show tumour infiltrating neutrophils promote AR expression in bladder cancer and RCC cells, which increases their metastatic potential (179, 180). Therefore, systemic administration of antiandrogens often shows equivocal effects on neutrophil-mediated tumour control.

#### 5.2.4 AR signalling in CAFs and endothelial cells

Several studies have demonstrated the important role of AR in preventing fibroblasts from differentiating into CAFs in skin cancers (181, 182) and prostate cancer (183–186). Low AR levels in prostate cancer stroma is associated with poorer patient survival. AR inhibits ANKRD1 (181) and LMO2 (183) expression, both of which are activators of CAF-related gene signatures. AR downregulation or deactivation leads to transition from normal fibroblasts to CAFs, enhancing tumorigenesis, tumour cell stemness and invasion via ECM remodelling and increased MMP expression (186), as well as increased expression of cytokines including IL-6, IL-8, IL-11, CCL2, IFNγ and M-CSF, all of which are also known to induce an immunosuppressive TME (182–185).

Further studies are anticipated on the effects of AR signalling in tumour endothelium and angiogenesis (187). While AR signalling in prostate cancer and RCC cells is known to upregulate angiogenic cytokines including VEGF and CXCL5 (188–190), AR signalling on endothelial cells themselves can also directly increase proliferation (191). AR-deficient or AR-antagonised endothelial cells show reduced angiogenic capacity and failure to activate eNOS (192, 193). How these findings may translate into tangible clinical intervention remains to be elucidated.

### 5.3 Mechanisms of AR and I/O resistance

Patient scRNA-seq and murine models have suggested that an increased AR signalling may predict I/O resistance, resulting from downregulation of IFNγ and upregulation of CD8^+^ T cell exhaustion programmes. Indeed, as previous sections have shown, a recurrent *in vivo* finding is that castration or T cell-specific AR knockout can improve I/O response in male mice, while antiandrogens rescue I/O response and tumour control in androgen-exposed females. While it is natural to test I/O-antiandrogen combinations in the clinical setting, I/O nevertheless fails to synergise with AR antagonists in metastatic CRPC after all, as evident in the IMbassador250 trial (194). Why is this?

One explanation, as discussed earlier, is that male CD8^+^ T cells have experienced long-term androgen exposure, predisposing them towards exhausted phenotypes during tumour progression, irrespective of subsequent AR signalling manipulation. In preclinical studies, castration or cell-specific AR knockout is almost always performed *before* tumour inoculation and I/O treatment. The dynamics of interaction between malignant cells and the TME may well be different from research involving antiandrogens. It reminds us that the sequence of I/O versus AR signalling manipulation is crucial to an optimised patient response.

Another hypothesis is that AR antagonists suppress anti-tumour immunity independently of AR. One study has shown that AR antagonists inhibit initial T cell priming via an off-target effect on GABA-A (195). Even if T cell exhaustion may be reduced with antiandrogen treatment, the initial neoantigen presentation and infiltration into the tumours can also be compromised, cancelling out the beneficial effect of AR antagonist on checkpoint inhibition. Indeed, another study also shows increased monocytic MDSC infiltration, decreased CD8+ TIL number and increased PD-L1 expression in enzalutamide-treated murine Myc-CaP tumours (168). When these tumour cells acquire enzalutamide resistance, they upregulate PD-L1 expression and possess an increased capacity to skew myeloid cells towards MDSCs and M2 macrophages (168, 195), further suppressing T cell function. Strikingly, another study shows a signalling crosstalk between AR and the glucocorticoid receptor (GR) (196). AR inhibition upregulates GR while high-dose steroids confer enzalutamide resistance to a prostate cancer model (196). This finding necessitates a more thorough understanding of the escape mechanisms of tumour cells when treated with combined I/O and antiandrogen (101).

Also importantly, as evident in previous sections, AR signalling exhibits heterogeneous effects on different TME cell types, resulting in equivocal efficacy when combining I/O with systemic antiandrogen administration. While AR on lymphocytes (T, B, NK) negatively regulates their cytotoxic functions in general, AR on macrophages and neutrophils regulate their functions in a sequence-dependent manner. Specifically, AR promotes the proliferation, maturation and infiltration of macrophages and neutrophils into the tissues. However, it subsequently renders these cells anti-inflammatory in the TME. AR inhibition also enhances the immunosuppressive functions of DCs and MDSCs. Furthermore, increasing evidence has shown that AR prevents fibroblast differentiation towards CAFs and regulates endothelial cell proliferation. Therefore, there is much unknown as to how systemic AR inhibition on a heterogenous TME affect immunotherapy efficacy. Interestingly, a recent analysis of NSCLC exosome and transcriptome datasets show significant enrichment of DCs and T cells as well as a T cell dysfunction phenotype in the TME of female patients, while the male patients generally possess a T cell excluded TME (197). These findings are highly consistent with the effects of AR signalling on TME cell types as described earlier, demonstrating the key role of AR in regulating tumour immunology and I/O response. Future combinatory I/O with AR modulation will require delicate consideration into the individual tumour characteristics.

Specifically in prostate cancer, preclinical studies have shown, as discussed above, how blocking AR signalling can in fact compromise T cell priming or activation (195), upregulate CCL2/STAT3-mediated macrophage recruitment (163), reduce neutrophil maturation or expansion (174), promote CAF accumulation (184), and increasing tumour cell expression of GR (196), all of which may negate the benefits of checkpoint blockade in these patients (198). Future combinatory trials in advanced prostate cancer will need to select patients early in their disease progression, and give careful thoughts on both the checkpoint (PD-1, PD-L1, CTLA4, TIGIT, etc.) to be targeted, as well as the timing of I/O relative to AR inhibition (198, 199).

## 6 Concluding remarks

The perceived sexual dimorphism in cancer epidemiology is the consequence of a myriad of factors, including socioeconomic and cultural disparities (200), environmental exposures, sex chromosomes, sex hormones, as well as sex hormone receptors such as AR. Indeed, gender oncology is emerging as an important aspect of personalised medicine that recognises and addresses such differences in cancer incidence and therapeutic responses (147). While research has elucidated the role of AR in tumour development and progression, studies have often overlooked the impact of AR signalling on the TME and I/O outcomes. We have shown that AR plays heterogeneous roles in individual TME cell types, sometimes independent of androgens, which potentially explains the equivocal efficacy of antiandrogen and I/O combination so far. It is hoped that future clinical studies on cancers could disaggregate outcomes by sex and stratify androgen/AR level more frequently, hence providing further evidence for antiandrogen and I/O combination or personalised I/O tailored to sex and androgen/AR status. Translational studies on AR modulation of the TME can help design better trials of I/O-based gender oncology with AR as a potential biomarker. By doing so we may optimise treatment strategies and improve individualised patient outcomes.

## Author contributions

JZ: Conceptualization, Data curation, Visualization, Writing – original draft, Writing – review & editing. QW: Conceptualization, Data curation, Visualization, Writing – original draft, Writing – review & editing. AT: Conceptualization, Data curation, Writing – original draft, Writing – review & editing. CL: Data curation, Visualization, Writing – original draft, Writing – review & editing. HT: Conceptualization, Resources, Supervision, Writing – review & editing, Writing – original draft.

## References

[1] Haupt S Caramia F Klein SL Rubin JB Haupt Y. Sex disparities matter in cancer development and therapy. Nat Rev Cancer. (2021) 21:393–407. doi: 10.1038/s41568-021-00348-y 33879867 PMC8284191

[2] Clayton JA Collins FS. Policy: NIH to balance sex in cell and animal studies. Nature. (2014) 509:282–3. doi: 10.1038/509282a PMC5101948 24834516

[3] Kajihara N Ge Y Seino K-I. Blocking of oestrogen signals improves anti-tumour effect regardless of oestrogen receptor alpha expression in cancer cells. Br J Cancer. (2023) 129:935–46. doi: 10.1038/s41416-023-02381-0 PMC10491758 37537255

[4] Yang C Jin J Yang Y Sun H Wu L Shen M. Androgen receptor-mediated CD8+ T cell stemness programs drive sex differences in antitumor immunity. Immunity. (2022) 55:1268–83.e9. doi: 10.1016/j.immuni.2022.05.012 35700739

[5] Bhatia A Sekhon HK Kaur G. Sex hormones and immune dimorphism. ScientificWorldJournal. (2014) 2014:159150. doi: 10.1155/2014/159150 25478584 PMC4251360

[6] Klein SL Flanagan KL. Sex differences in immune responses. Nat Rev Immunol. (2016) 16:626–38. doi: 10.1038/nri.2016.90 27546235

[7] Whitacre CC. Sex differences in autoimmune disease. Nat Immunol. (2001) 2:777–80. doi: 10.1038/ni0901-777 11526384

[8] Lockshin MD. Sex differencesxxx inxxx autoimmunexxxx disease. Lupus. (2006) 15:753–6. doi: 10.1177/0961203306069353 17153846

[9] Henze L Schwinge D Schramm C. The effects of androgens on T cells: clues to female predominance in autoimmune liver diseases? Front Immunol. (2020) 11:1567. doi: 10.3389/fimmu.2020.01567 32849531 PMC7403493

[10] Colafella KMM Denton KM. Sex-specific differences in hypertension and associated cardiovascular disease. Nat Rev Nephrol. (2018) 14:185–201. doi: 10.1038/nrneph.2017.189 29380817

[11] Shobab L Burman KD Wartofsky L. Sex differences in differentiated thyroid cancer. Thyroid. (2022) 32:224–35. doi: 10.1089/thy.2021.0361 34969307

[12] Edgren G Liang L Adami H-O Chang ET. Enigmatic sex disparities in cancer incidence. Eur J Epidemiol. (2012) 27:187–96. doi: 10.1007/s10654-011-9647-5 22212865

[13] Sung H Ferlay J Siegel RL Laversanne M Soerjomataram I Jemal A. Global cancer statistics 2020: GLOBOCAN estimates of incidence and mortality worldwide for 36 cancers in 185 countries. CA Cancer J Clin. (2021) 71:209–49. doi: 10.3322/caac.21660 33538338

[14] Ferlay J Ervik M Lam F Laversanne M Colombet M Mery L. Global Cancer Observatory: Cancer Today (version 1.0). Lyon, France: International Agency for Research on Cancer. (2020). Available at: https://gco.iarc.who.int/today (Accessed January 10, 2024).

[15] Zahedi A Bondaz L Rajaraman M Leslie WD Jefford C Young JE. Risk for thyroid cancer recurrence is higher in men than in women independent of disease stage at presentation. Thyroid. (2020) 30:871–7. doi: 10.1089/thy.2018.0775 31524071

[16] Mancini M Righetto M Baggio G. Spotlight on gender-specific disparities in bladder cancer. Urologia. (2020) 87:103–14. doi: 10.1177/0391560319887327 31868559

[17] Siegel RL Miller KD Wagle NS Jemal A. Cancer statistics, 2023. CA Cancer J Clin. (2023) 73:17–48. doi: 10.3322/caac.21763 36633525

[18] Li Q Cao M Lei L Yang F Li H Yan X. Burden of liver cancer: From epidemiology to prevention. Chin J Cancer Res. (2022) 34:554–66. doi: 10.21147/j.issn.1000-9604.2022.06.02 PMC9829497 36714347

[19] Thrift AP Wenker TN El-Serag HB. Global burden of gastric cancer: epidemiological trends, risk factors, screening and prevention. Nat Rev Clin Oncol. (2023) 20:338–49. doi: 10.1038/s41571-023-00747-0 36959359

[20] Siegel RL Miller KD Fuchs HE Jemal A. Cancer statistics, 2021. CA Cancer J Clin. (2021) 71:7–33. doi: 10.3322/caac.21654 33433946

[21] Martínez-Rojo E Berumen LC García-Alcocer G Escobar-Cabrera J. The role of androgens and androgen receptor in human bladder cancer. Biomolecules. (2021) 11(4):594. doi: 10.3390/biom11040594 33919565 PMC8072960

[22] Bell DW Brannigan BW Matsuo K Finkelstein DM Sordella R Settleman J. Increased prevalence of EGFR-mutant lung cancer in women and in East Asian populations: analysis of estrogen-related polymorphisms. Clin Cancer Res. (2008) 14:4079–84. doi: 10.1158/1078-0432.CCR-07-5030 PMC3391698 18593984

[23] Jemal A Schafer EJ Sung H Bandi P Kratzer T Islami F. The burden of lung cancer in women compared with men in the US. JAMA Oncol. (2023) 9:1727–8. doi: 10.1001/jamaoncol.2023.4415 PMC10570912 37824139

[24] Xie S-H Yu IT-S Tse L-A Mang OW-K Yue L. Sex difference in the incidence of nasopharyngeal carcinoma in Hong Kong 1983–2008: suggestion of a potential protective role of oestrogen. Eur J Cancer. (2013) 49:150–5. doi: 10.1016/j.ejca.2012.07.004 22892061

[25] Arnold M Singh D Laversanne M Vignat J Vaccarella S Meheus F. Global burden of cutaneous melanoma in 2020 and projections to 2040. JAMA Dermatol. (2022) 158:495–503. doi: 10.1001/jamadermatol.2022.0160 35353115 PMC8968696

[26] Scoggins CR Ross MI Reintgen DS Noyes RD Goydos JS Beitsch PD. Gender-related differences in outcome for melanoma patients. Ann Surg. (2006) 243:693–8; discussion 698–700. doi: 10.1097/01.sla.0000216771.81362.6b 16633005 PMC1570554

[27] Manola J Atkins M Ibrahim J Kirkwood J. Prognostic factors in metastatic melanoma: a pooled analysis of Eastern Cooperative Oncology Group trials. J Clin Oncol. (2000) 18:3782–93. doi: 10.1200/JCO.2000.18.22.3782 11078491

[28] Nosrati A Wei ML. Sex disparities in melanoma outcomes: the role of biology. Arch Biochem Biophys. (2014) 563:42–50. doi: 10.1016/j.abb.2014.06.018 25057772

[29] Dong M Cioffi G Wang J Waite KA Ostrom QT Kruchko C. Sex differences in cancer incidence and survival: A pan-cancer analysis. Cancer Epidemiol Biomarkers Prev. (2020) 29:1389–97. doi: 10.1158/1055-9965.EPI-20-0036 32349967

[30] Brown KF Rumgay H Dunlop C Ryan M Quartly F Cox A. The fraction of cancer attributable to modifiable risk factors in England, Wales, Scotland, Northern Ireland, and the United Kingdom in 2015. Br J Cancer. (2018) 118:1130–41. doi: 10.1038/s41416-018-0029-6 PMC5931106 29567982

[31] Bryant A Cerfolio RJ. Differences in epidemiology, histology, and survival between cigarette smokers and never-smokers who develop non-small cell lung cancer. Chest. (2007) 132:185–92. doi: 10.1378/chest.07-0442 17573517

[32] Morgan TR Mandayam S Jamal MM. Alcohol and hepatocellular carcinoma. Gastroenterology. (2004) 127:S87–96. doi: 10.1053/j.gastro.2004.09.020 15508108

[33] Corrao G Bagnardi V Zambon A La Vecchia C. A meta-analysis of alcohol consumption and the risk of 15 diseases. Prev Med. (2004) 38:613–9. doi: 10.1016/j.ypmed.2003.11.027 15066364

[34] Cho E Smith-Warner SA Ritz J van den Brandt PA Colditz GA Folsom AR. Alcohol intake and colorectal cancer: a pooled analysis of 8 cohort studies. Ann Intern Med. (2004) 140:603–13. doi: 10.7326/0003-4819-140-8-200404200-00007 15096331

[35] Verplaetse TL Peltier MR Roberts W Burke C Moore KE Pittman B. Sex and alcohol use disorder predict the presence of cancer, respiratory, and other medical conditions: Findings from the National Epidemiologic Survey on Alcohol and Related Conditions-III. Addict Behav. (2021) 123:107055. doi: 10.1016/j.addbeh.2021.107055 34311184 PMC8419091

[36] Hydes TJ Burton R Inskip H Bellis MA Sheron N. A comparison of gender-linked population cancer risks between alcohol and tobacco: how many cigarettes are there in a bottle of wine? BMC Public Health. (2019) 19:316. doi: 10.1186/s12889-019-6576-9 30917803 PMC6437970

[37] Steele CB Thomas CC Henley SJ Massetti GM Galuska DA Agurs-Collins T. Vital signs: trends in incidence of cancers associated with overweight and obesity - United States, 2005–2014. MMWR Morb Mortal Wkly Rep. (2017) 66:1052–8. doi: 10.15585/mmwr.mm6639e1 PMC5720881 28981482

[38] Arnold M Soerjomataram I Ferlay J Forman D. Global incidence of oesophageal cancer by histological subtype in 2012. Gut. (2015) 64:381–7. doi: 10.1136/gutjnl-2014-308124 25320104

[39] Argyrakopoulou G Dalamaga M Spyrou N Kokkinos A. Gender differences in obesity-related cancers. Curr Obes Rep. (2021) 10:100–15. doi: 10.1007/s13679-021-00426-0 33523397

[40] Avgerinos KI Spyrou N Mantzoros CS Dalamaga M. Obesity and cancer risk: Emerging biological mechanisms and perspectives. Metabolism. (2019) 92:121–35. doi: 10.1016/j.metabol.2018.11.001 30445141

[41] Lee CH Woo YC Wang Y Yeung CY Xu A Lam KSL. Obesity, adipokines and cancer: an update. Clin Endocrinol. (2015) 83:147–56. doi: 10.1111/cen.12667 25393563

[42] Ballestri S Nascimbeni F Baldelli E Marrazzo A Romagnoli D Lonardo A. NAFLD as a sexual dimorphic disease: role of gender and reproductive status in the development and progression of nonalcoholic fatty liver disease and inherent cardiovascular risk. Adv Ther. (2017) 34:1291–326. doi: 10.1007/s12325-017-0556-1 PMC5487879 28526997

[43] Elkhalifa AME Nabi SU Shah OS Bashir SM Muzaffer U Ali SI. Current Oncology. Available at: https://www.mdpi.com/1718–7729/30/2/150/review_report (Accessed February 27, 2024).

[44] Mundi N Ghasemi F Zeng PYF Prokopec SD Patel K Kim HAJ. Sex disparities in head & neck cancer driver genes: An analysis of the TCGA dataset. Oral Oncol. (2020) 104:104614. doi: 10.1016/j.oraloncology.2020.104614 32146388

[45] El-Serag HB Rudolph KL. Hepatocellular carcinoma: epidemiology and molecular carcinogenesis. Gastroenterology. (2007) 132:2557–76. doi: 10.1053/j.gastro.2007.04.061 17570226

[46] Tukiainen T Villani A-C Yen A Rivas MA Marshall JL Satija R. Landscape of X chromosome inactivation across human tissues. Nature. (2017) 550:244–8. doi: 10.1038/nature24265 PMC5685192 29022598

[47] Dunford A Weinstock DM Savova V Schumacher SE Cleary JP Yoda A. Tumor-suppressor genes that escape from X-inactivation contribute to cancer sex bias. Nat Genet. (2017) 49:10–6. doi: 10.1038/ng.3726 PMC5206905 27869828

[48] Zito A Davies MN Tsai P-C Roberts S Andres-Ejarque R Nardone S. Heritability of skewed X-inactivation in female twins is tissue-specific and associated with age. Nat Commun. (2019) 10:5339. doi: 10.1038/s41467-019-13340-w 31767861 PMC6877649

[49] Haupt S Caramia F Herschtal A Soussi T Lozano G Chen H. Identification of cancer sex-disparity in the functional integrity of p53 and its X chromosome network. Nat Commun. (2019) 10:5385. doi: 10.1038/s41467-019-13266-3 31772231 PMC6879765

[50] Büscheck F Fraune C Garmestani S Simon R Kluth M Hube-Magg C. Y-chromosome loss is frequent in male renal tumors. Ann Transl Med. (2021) 9:209. doi: 10.21037/atm 33708836 PMC7940894

[51] Willis-Owen SAG Domingo-Sabugo C Starren E Liang L Freidin MB Arseneault M. Y disruption, autosomal hypomethylation and poor male lung cancer survival. Sci Rep. (2021) 11:12453. doi: 10.1038/s41598-021-91907-8 34127738 PMC8203787

[52] Abdel-Hafiz HA Schafer JM Chen X Xiao T Gauntner TD Li Z. Y chromosome loss in cancer drives growth by evasion of adaptive immunity. Nature. (2023) 619:624–31. doi: 10.1038/s41586-023-06234-x PMC10975863 37344596

[53] Cáceres A Jene A Esko T Pérez-Jurado LA González JR. Extreme downregulation of chromosome Y and cancer risk in men. J Natl Cancer Inst. (2020) 112:913–20. doi: 10.1093/jnci/djz232 PMC7492764 31945786

[54] Kool M Koster J Bunt J Hasselt NE Lakeman A van Sluis P. Integrated genomics identifies five medulloblastoma subtypes with distinct genetic profiles, pathway signatures and clinicopathological features. PloS One. (2008) 3:e3088. doi: 10.1371/journal.pone.0003088 18769486 PMC2518524

[55] Parodi S Pistorio A Erminio G Ognibene M Morini M Garaventa A. Loss of whole chromosome X predicts prognosis of neuroblastoma patients with numerical genomic profile. Pediatr Blood Cancer. (2019) 66:e27635. doi: 10.1002/pbc.27635 30688024

[56] Duijf PHG Schultz N Benezra R. Cancer cells preferentially lose small chromosomes. Int J Cancer. (2013) 132:2316–26. doi: 10.1002/ijc.27924 PMC3587043 23124507

[57] Shuai C Yang X Pan H Han W. Estrogen receptor downregulates expression of PD-1/PD-L1 and infiltration of CD8+ T cells by inhibiting IL-17 signaling transduction in breast cancer. Front Oncol. (2020) 10:582863. doi: 10.3389/fonc.2020.582863 33102239 PMC7545792

[58] Kang C Song C-H Kim N Nam RH Choi SI Yu JE. The enhanced inhibitory effect of estrogen on PD-L1 expression following Nrf2 deficiency in the AOM/DSS model of colitis-associated cancer. Front Oncol. (2021) 11:679324. doi: 10.3389/fonc.2021.679324 34307147 PMC8297827

[59] Liu S Fan W Gao X Huang K Ding C Ma G. Estrogen receptor alpha regulates the Wnt/β-catenin signaling pathway in colon cancer by targeting the NOD-like receptors. Cell Signal. (2019) 61:86–92. doi: 10.1016/j.cellsig.2019.05.009 31121307

[60] Clusan L Ferrière F Flouriot G Pakdel F. A basic review on estrogen receptor signaling pathways in breast cancer. Int J Mol Sci. (2023) 24(7):6834. doi: 10.3390/ijms24076834 37047814 PMC10095386

[61] McGlynn LM Tovey S Bartlett JMS Doughty J Cooke TG Edwards J. Interactions between MAP kinase and oestrogen receptor in human breast cancer. Eur J Cancer. (2013) 49:1176–86. doi: 10.1016/j.ejca.2012.11.020 23265704

[62] Kouzmenko AP Takeyama K-I Ito S Furutani T Sawatsubashi S Maki A. Wnt/beta-catenin and estrogen signaling converge in vivo. J Biol Chem. (2004) 279:40255–8. doi: 10.1074/jbc.C400331200 15304487

[63] Niv Y. Estrogen receptor β expression and colorectal cancer: a systematic review and meta-analysis. Eur J Gastroenterol Hepatol. (2015) 27:1438–42. doi: 10.1097/MEG.0000000000000471 26367493

[64] Rossouw JE Anderson GL Prentice RL LaCroix AZ Kooperberg C Stefanick ML. Risks and benefits of estrogen plus progestin in healthy postmenopausal women: principal results From the Women’s Health Initiative randomized controlled trial. JAMA. (2002) 288:321–33. doi: 10.1001/jama.288.3.321 12117397

[65] Rawłuszko-Wieczorek AA Lipowicz J Nowacka M Ostrowska K Pietras P Blatkiewicz M. Estrogen receptor β affects hypoxia response in colorectal cancer cells. Biochim Biophys Acta Mol Basis Dis. (2024) 1870:166894. doi: 10.1016/j.bbadis.2023.166894 37748565

[66] Bravo-Vázquez LA Méndez-García A Rodríguez AL Sahare P Pathak S Banerjee A. Applications of nanotechnologies for miRNA-based cancer therapeutics: current advances and future perspectives. Front Bioeng Biotechnol. (2023) 11:1208547. doi: 10.3389/fbioe.2023.1208547 37576994 PMC10416113

[67] Pang C Li J-M Wang Z Luo Y-C Cheng Z-G Han Z-Y. Age-dependent female survival advantage in hepatocellular carcinoma: A multicenter cohort study. Clin Gastroenterol Hepatol. (2024) 22:305–14. doi: 10.1016/j.cgh.2023.07.029 37659766

[68] Balcar L Scheiner B Fulgenzi CAM D’Alessio A Pomej K Roig MB. A meta-analysis and real-world cohort study on the sex-related differences in efficacy and safety of immunotherapy for hepatocellular carcinoma. JHEP Rep. (2024) 6:100982. doi: 10.1016/j.jhepr.2023.100982 38274490 PMC10809085

[69] Farinati F Salvagnini M de Maria N Fornasiero A Chiaramonte M Rossaro L. Unresectable hepatocellular carcinoma: a prospective controlled trial with tamoxifen. J Hepatol. (1990) 11:297–301. doi: 10.1016/0168-8278(90)90211-9 1705274

[70] Perrone F Gallo C Daniele B Gaeta GB Izzo F Capuano G. Tamoxifen in the treatment of hepatocellular carcinoma: 5-year results of the CLIP-1 multicentre randomised controlled trial. Curr Pharm Des. (2002) 8:1013–9. doi: 10.2174/1381612024607063 11945148

[71] Castells A Bruix J Brú C Ayuso C Roca M Boix L. Treatment of hepatocellular carcinoma with tamoxifen: a double-blind placebo-controlled trial in 120 patients. Gastroenterology. (1995) 109:917–22. doi: 10.1016/0016-5085(95)90402-6 7657122

[72] Zeng H Yang Z Li J Wen Y Wu Z Zheng Y. Associations between female lung cancer risk and sex steroid hormones: a systematic review and meta-analysis of the worldwide epidemiological evidence on endogenous and exogenous sex steroid hormones. BMC Cancer. (2021) 21:690. doi: 10.1186/s12885-021-08437-9 34112140 PMC8194027

[73] Castellanos MR Fanous E Thaker R Flory MJ Seetharamu N Dhar M. Expression patterns and clinical significance of estrogen receptor in non-small cell lung cancer. Pathol Res Pract. (2023) 241:154298. doi: 10.1016/j.prp.2022.154298 36608623

[74] Kawai H Ishii A Washiya K Konno T Kon H Yamaya C. Estrogen receptor alpha and beta are prognostic factors in non-small cell lung cancer. Clin Cancer Res. (2005) 11:5084–9. doi: 10.1158/1078-0432.CCR-05-0200 16033821

[75] Li H Chen H Shi J Fan Q Zhou Z Tang X. ERβ overexpression may not be a direct prognostic factor in patients with NSCLC: A meta-analysis. Int J Biol Markers. (2022) 37:249–59. doi: 10.1177/03936155221105521 35730164

[76] Meng W Liao Y Chen J Wang Y Meng Y Li K. Upregulation of estrogen receptor beta protein but not mRNA predicts poor prognosis and may be associated with enhanced translation in non-small cell lung cancer: a systematic review and meta-analysis. J Thorac Dis. (2021) 13:4281–300. doi: 10.21037/jtd PMC8339768 34422356

[77] Cronin-Fenton DP Murray LJ Whiteman DC Cardwell C Webb PM Jordan SJ. Reproductive and sex hormonal factors and oesophageal and gastric junction adenocarcinoma: a pooled analysis. Eur J Cancer. (2010) 46:2067–76. doi: 10.1016/j.ejca.2010.03.032 PMC5542412 20456945

[78] Camargo MC Goto Y Zabaleta J Morgan DR Correa P Rabkin CS. Sex hormones, hormonal interventions, and gastric cancer risk: a meta-analysis. Cancer Epidemiol Biomarkers Prev. (2012) 21:20–38. doi: 10.1158/1055-9965.EPI-11-0834 22028402 PMC3315355

[79] Raynor MC Carson CC Pearson MD Nix JW. Androgen deficiency in the aging male: a guide to diagnosis and testosterone replacement therapy. Can J Urol. (2007) 14 Suppl 1:63–8. 18163948

[80] Iwasa T Yamamoto Y Shinya A Minato S Yanagihara R Kamada S. The effects of androgens on metabolic functions in females. J Med Invest. (2021) 68:228–31. doi: 10.2152/jmi.68.228 34759135

[81] Liu Z Zhang Y Lagergren J Li S Li J Zhou Z. Circulating sex hormone levels and risk of gastrointestinal cancer: systematic review and meta-analysis of prospective studies. Cancer Epidemiol Biomarkers Prev. (2023) 32:936–46. doi: 10.1158/1055-9965.EPI-23-0039 37104672

[82] Xie S-H Ness-Jensen E Rabbani S Langseth H Gislefoss RE Mattsson F. Circulating sex hormone levels and risk of esophageal adenocarcinoma in a prospective study in men. Am J Gastroenterol. (2020) 115:216–23. doi: 10.14309/ajg.0000000000000446 31658123

[83] Garg H Wheeler KM Dursun F Cooper RE Pruthi DK Kaushik D. Impact of finasteride on survival in bladder cancer: A retrospective multi-institutional database analysis. Clin Genitourin Cancer. (2023) 21:314.e1–7. doi: 10.1016/j.clgc.2022.10.014 36402643

[84] Edelsztein NY Rey RA. Importance of the androgen receptor signaling in gene transactivation and transrepression for pubertal maturation of the testis. Cells. (2019) 8(8):861. doi: 10.3390/cells8080861 31404977 PMC6721648

[85] Pietri E Conteduca V Andreis D Massa I Melegari E Sarti S. Androgen receptor signaling pathways as a target for breast cancer treatment. Endocr Relat Cancer. (2016) 23:R485–98. doi: 10.1530/ERC-16-0190 27528625

[86] Bennett NC Gardiner RA Hooper JD Johnson DW Gobe GC. Molecular cell biology of androgen receptor signalling. Int J Biochem Cell Biol. (2010) 42:813–27. doi: 10.1016/j.biocel.2009.11.013 19931639

[87] Kanda T Jiang X Yokosuka O. Androgen receptor signaling in hepatocellular carcinoma and pancreatic cancers. World J Gastroenterol. (2014) 20:9229–36. doi: 10.3748/wjg.v20.i28.9229 PMC4110552 25071315

[88] Rothman MS Carlson NE Xu M Wang C Swerdloff R Lee P. Reexamination of testosterone, dihydrotestosterone, estradiol and estrone levels across the menstrual cycle and in postmenopausal women measured by liquid chromatography-tandem mass spectrometry. Steroids. (2011) 76:177–82. doi: 10.1016/j.steroids.2010.10.010 PMC3005029 21070796

[89] Cooke PS Walker WH. Nonclassical androgen and estrogen signaling is essential for normal spermatogenesis. Semin Cell Dev Biol. (2022) 121:71–81. doi: 10.1016/j.semcdb.2021.05.032 34119408

[90] Gibson DA Saunders PTK McEwan IJ. Androgens and androgen receptor: Above and beyond. Mol Cell Endocrinol. (2018) 465:1–3. doi: 10.1016/j.mce.2018.02.013 29481861

[91] Becerra-Diaz M Song M Heller N. Androgen and androgen receptors as regulators of monocyte and macrophage biology in the healthy and diseased lung. Front Immunol. (2020) 11:1698. doi: 10.3389/fimmu.2020.01698 32849595 PMC7426504

[92] Mantalaris A Panoskaltsis N Sakai Y Bourne P Chang C Messing EM. Localization of androgen receptor expression in human bone marrow. J Pathol. (2001) 193:361–6. doi: 10.1002/(ISSN)1096-9896 11241417

[93] Sinnesael M Boonen S Claessens F Gielen E Vanderschueren D. Testosterone and the male skeleton: a dual mode of action. J Osteoporos. (2011) 2011:240328. doi: 10.4061/2011/240328 21941679 PMC3173882

[94] Swerdloff RS Dudley RE Page ST Wang C Salameh WA. Dihydrotestosterone: biochemistry, physiology, and clinical implications of elevated blood levels. Endocr Rev. (2017) 38:220–54. doi: 10.1210/er.2016-1067 PMC6459338 28472278

[95] Lang F Alevizopoulos K Stournaras C. Targeting membrane androgen receptors in tumors. Expert Opin Ther Targets. (2013) 17:951–63. doi: 10.1517/14728222.2013.806491 23746222

[96] Papakonstanti EA Kampa M Castanas E Stournaras C. A rapid, nongenomic, signaling pathway regulates the actin reorganization induced by activation of membrane testosterone receptors. Mol Endocrinol. (2003) 17:870–81. doi: 10.1210/me.2002-0253 12554777

[97] Lin AJ Baranski T Chaterjee D Chapman W Foltz G Kim H. Androgen-receptor-positive hepatocellular carcinoma in a transgender teenager taking exogenous testosterone. Lancet. (2020) 396:198. doi: 10.1016/S0140-6736(20)31538-5 32682485

[98] Caroppo F Tadiotto Cicogna G Messina F Alaibac M. Association between melanoma and exposure to sex hormones in puberty: A possible window of susceptibility (Review). Mol Clin Oncol. (2021) 14:66. doi: 10.3892/mco 33680457 PMC7890437

[99] Rampen FH Mulder JH. Malignant melanoma: an androgen-dependent tumour? Lancet. (1980) 1:562–4. doi: 10.1016/S0140-6736(80)91055-7 6102285

[100] Pothuri VS Anzelmo M Gallaher E Ogunlana Y Aliabadi-Wahle S Tan B. Transgender males on gender-affirming hormone therapy and hepatobiliary neoplasms: A systematic review. Endocr Pract. (2023) 29:822–9. doi: 10.1016/j.eprac.2023.05.011 37286102

[101] Watson PA Arora VK Sawyers CL. Emerging mechanisms of resistance to androgen receptor inhibitors in prostate cancer. Nat Rev Cancer. (2015) 15:701–11. doi: 10.1038/nrc4016 PMC4771416 26563462

[102] Schweizer MT Yu EY. AR-signaling in human Malignancies: prostate cancer and beyond. Cancers. (2017) 9(1):7. doi: 10.3390/cancers9010007 28085048 PMC5295778

[103] Scher HI Fizazi K Saad F Taplin M-E Sternberg CN Miller K. Increased survival with enzalutamide in prostate cancer after chemotherapy. N Engl J Med. (2012) 367:1187–97. doi: 10.1056/NEJMoa1207506 22894553

[104] Wang Q Li W Liu XS Carroll JS Jänne OA Keeton EK. A hierarchical network of transcription factors governs androgen receptor-dependent prostate cancer growth. Mol Cell. (2007) 27:380–92. doi: 10.1016/j.molcel.2007.05.041 PMC3947890 17679089

[105] Peters AA Buchanan G Ricciardelli C Bianco-Miotto T Centenera MM Harris JM. Androgen receptor inhibits estrogen receptor-alpha activity and is prognostic in breast cancer. Cancer Res. (2009) 69:6131–40. doi: 10.1158/0008-5472.CAN-09-0452 19638585

[106] Lehmann BD Bauer JA Chen X Sanders ME Chakravarthy AB Shyr Y. Identification of human triple-negative breast cancer subtypes and preclinical models for selection of targeted therapies. J Clin Invest. (2011) 121:2750–67. doi: 10.1172/JCI45014 PMC3127435 21633166

[107] Asemota S Effah W Young KL Holt J Cripe L Ponnusamy S. Identification of a targetable JAK-STAT enriched androgen receptor and androgen receptor splice variant positive triple-negative breast cancer subtype. Cell Rep. (2023) 42:113461. doi: 10.1016/j.celrep.2023.113461 37979170 PMC10872270

[108] Lehmann BD Bauer JA Schafer JM Pendleton CS Tang L Johnson KC. PIK3CA mutations in androgen receptor-positive triple negative breast cancer confer sensitivity to the combination of PI3K and androgen receptor inhibitors. Breast Cancer Res. (2014) 16:406. doi: 10.1186/s13058-014-0406-x 25103565 PMC4187324

[109] Luk PP Weston JD Yu B Selinger CI Ekmejian R Eviston TJ. Salivary duct carcinoma: Clinicopathologic features, morphologic spectrum, and somatic mutations. Head Neck. (2016) 38 Suppl 1:E1838–47. doi: 10.1002/hed.24332 26699379

[110] Williams L Thompson LDR Seethala RR Weinreb I Assaad AM Tuluc M. Salivary duct carcinoma: the predominance of apocrine morphology, prevalence of histologic variants, and androgen receptor expression. Am J Surg Pathol. (2015) 39:705–13. doi: 10.1097/PAS.0000000000000413 25871467

[111] Nasser SM Faquin WC Dayal Y. Expression of androgen, estrogen, and progesterone receptors in salivary gland tumors. Frequent expression of androgen receptor in a subset of Malignant salivary gland tumors. Am J Clin Pathol. (2003) 119:801–6. doi: 10.1309/RVTP1G0Q727WJUQD 12817426

[112] Mitani Y Rao PH Maity SN Lee Y-C Ferrarotto R Post JC. Alterations associated with androgen receptor gene activation in salivary duct carcinoma of both sexes: potential therapeutic ramifications. Clin Cancer Res. (2014) 20:6570–81. doi: 10.1158/1078-0432.CCR-14-1746 PMC4268116 25316813

[113] Dalin MG Desrichard A Katabi N Makarov V Walsh LA Lee K-W. Comprehensive molecular characterization of salivary duct carcinoma reveals actionable targets and similarity to apocrine breast cancer. Clin Cancer Res. (2016) 22:4623–33. doi: 10.1158/1078-0432.CCR-16-0637 PMC5026550 27103403

[114] Morova T McNeill DR Lallous N Gönen M Dalal K Wilson DM 3rd. Androgen receptor-binding sites are highly mutated in prostate cancer. Nat Commun. (2020) 11:832. doi: 10.1038/s41467-020-14644-y 32047165 PMC7012874

[115] Hodgkin J Zellan JD Albertson DG. Identification of a candidate primary sex determination locus, fox-1, on the X chromosome of Caenorhabditis elegans. Development. (1994) 120:3681–9. doi: 10.1242/dev.120.12.3681 7821230

[116] Jozwik KM Carroll JS. Pioneer factors in hormone-dependent cancers. Nat Rev Cancer. (2012) 12:381–5. doi: 10.1038/nrc3263 22555282

[117] Robinson JLL Macarthur S Ross-Innes CS Tilley WD Neal DE Mills IG. Androgen receptor driven transcription in molecular apocrine breast cancer is mediated by FoxA1. EMBO J. (2011) 30:3019–27. doi: 10.1038/emboj.2011.216 PMC3160190 21701558

[118] Barbieri CE Baca SC Lawrence MS Demichelis F Blattner M Theurillat J-P. Exome sequencing identifies recurrent SPOP, FOXA1 and MED12 mutations in prostate cancer. Nat Genet. (2012) 44:685–9. doi: 10.1038/ng.2279 PMC3673022 22610119

[119] Sahu B Laakso M Ovaska K Mirtti T Lundin J Rannikko A. Dual role of FoxA1 in androgen receptor binding to chromatin, androgen signalling and prostate cancer. EMBO J. (2011) 30:3962–76. doi: 10.1038/emboj.2011.328 PMC3209787 21915096

[120] Li Z Tuteja G Schug J Kaestner KH. Foxa1 and Foxa2 are essential for sexual dimorphism in liver cancer. Cell. (2012) 148:72–83. doi: 10.1016/j.cell.2011.11.026 22265403 PMC3266536

[121] Yang L Xie S Jamaluddin MS Altuwaijri S Ni J Kim E. Induction of androgen receptor expression by phosphatidylinositol 3-kinase/Akt downstream substrate, FOXO3a, and their roles in apoptosis of LNCaP prostate cancer cells. J Biol Chem. (2005) 280:33558–65. doi: 10.1074/jbc.M504461200 16061480

[122] Liu P Li S Gan L Kao TP Huang H. A transcription-independent function of FOXO1 in inhibition of androgen-independent activation of the androgen receptor in prostate cancer cells. Cancer Res. (2008) 68:10290–9. doi: 10.1158/0008-5472.CAN-08-2038 19074897

[123] Zheng Y Izumi K Yao JL Miyamoto H. Dihydrotestosterone upregulates the expression of epidermal growth factor receptor and ERBB2 in androgen receptor-positive bladder cancer cells. Endocr Relat Cancer. (2011) 18:451–64. doi: 10.1530/ERC-11-0010 21613411

[124] Li Y Zheng Y Izumi K Ishiguro H Ye B Li F. Androgen activates β-catenin signaling in bladder cancer cells. Endocr Relat Cancer. (2013) 20:293–304. doi: 10.1530/ERC-12-0328 23447569

[125] Lee E Madar A David G Garabedian MJ Dasgupta R Logan SK. Inhibition of androgen receptor and β-catenin activity in prostate cancer. Proc Natl Acad Sci U S A. (2013) 110:15710–5. doi: 10.1073/pnas.1218168110 PMC3785716 24019458

[126] Zhao X Shan Q Xue H-H. TCF1 in T cell immunity: a broadened frontier. Nat Rev Immunol. (2022) 22:147–57. doi: 10.1038/s41577-021-00563-6 34127847

[127] Vellano CP White MG Andrews MC Chelvanambi M Witt RG Daniele JR. Androgen receptor blockade promotes response to BRAF/MEK-targeted therapy. Nature. (2022) 606:797–803. doi: 10.1038/s41586-022-04833-8 35705814 PMC10071594

[128] Samarkina A Youssef MK Ostano P Ghosh S Ma M Tassone B. Androgen receptor is a determinant of melanoma targeted drug resistance. Nat Commun. (2023) 14:6498. doi: 10.1038/s41467-023-42239-w 37838724 PMC10576812

[129] Conforti F Pala L Bagnardi V De Pas T Martinetti M Viale G. Cancer immunotherapy efficacy and patients’ sex: a systematic review and meta-analysis. Lancet Oncol. (2018) 19:737–46. doi: 10.1016/S1470-2045(18)30261-4 29778737

[130] Conforti F Pala L Bagnardi V Viale G De Pas T Pagan E. Sex-based heterogeneity in response to lung cancer immunotherapy: A systematic review and meta-analysis. J Natl Cancer Inst. (2019) 111:772–81. doi: 10.1093/jnci/djz094 PMC6695312 31106827

[131] Wei Y Li Y Du Q Peng X Jin J Guo H. Effects of clinicopathological characteristics on the survival of patients treated with PD-1/PD-L1 inhibitor monotherapy or combination therapy for advanced cancer: A systemic review and meta-analysis. J Immunol Res. (2020) 2020:5269787. doi: 10.1155/2020/5269787 33381603 PMC7762667

[132] Takada K Shimokawa M Mizuki F Takamori S Takenaka T Miura N. Association between sex and outcomes in patients with non-small-cell lung cancer receiving combination chemoimmunotherapy as a first-line therapy: a systematic review and meta-analysis of randomized clinical trials. Eur J Med Res. (2022) 27:157. doi: 10.1186/s40001-022-00789-7 35999618 PMC9400263

[133] Yanagisawa T Kawada T Quhal F Bekku K Laukhtina E Rajwa P. Impact of sex on the efficacy of immune checkpoint inhibitors in kidney and urothelial cancers: a systematic review and meta-analysis. World J Urol. (2023) 41:1763–74. doi: 10.1007/s00345-023-04412-0 PMC10352444 37209143

[134] Shi Y Au JS-K Thongprasert S Srinivasan S Tsai C-M Khoa MT. A prospective, molecular epidemiology study of EGFR mutations in Asian patients with advanced non-small-cell lung cancer of adenocarcinoma histology (PIONEER). J Thorac Oncol. (2014) 9:154–62. doi: 10.1097/JTO.0000000000000033 PMC4132036 24419411

[135] Finn RS Qin S Ikeda M Galle PR Ducreux M Kim T-Y. Atezolizumab plus bevacizumab in unresectable hepatocellular carcinoma. N Engl J Med. (2020) 382:1894–905. doi: 10.1056/NEJMoa1915745 32402160

[136] Zhu AX Abbas AR de Galarreta MR Guan Y Lu S Koeppen H. Molecular correlates of clinical response and resistance to atezolizumab in combination with bevacizumab in advanced hepatocellular carcinoma. Nat Med. (2022) 28:1599–611. doi: 10.1038/s41591-022-01868-2 35739268

[137] Abou-Alfa GK Lau G Kudo M Chan SL Kelley RK Furuse J. Tremelimumab plus durvalumab in unresectable hepatocellular carcinoma. NEJM Evid. (2022) 1:EVIDoa2100070. doi: 10.1056/EVIDoa2100070 38319892

[138] Doki Y Ajani JA Kato K Xu J Wyrwicz L Motoyama S. Nivolumab combination therapy in advanced esophageal squamous-cell carcinoma. N Engl J Med. (2022) 386:449–62. doi: 10.1056/NEJMoa2111380 35108470

[139] André T Tougeron D Piessen G de la Fouchardière C Louvet C Adenis A. Neoadjuvant nivolumab plus ipilimumab and adjuvant nivolumab in localized deficient mismatch repair/microsatellite instability-high gastric or esophagogastric junction adenocarcinoma: the GERCOR NEONIPIGA phase II study. J Clin Oncol. (2023) 41:255–65. doi: 10.1200/JCO.22.00686 PMC9839243 35969830

[140] Bang Y-J Van Cutsem E Feyereislova A Chung HC Shen L Sawaki A. Trastuzumab in combination with chemotherapy versus chemotherapy alone for treatment of HER2-positive advanced gastric or gastro-oesophageal junction cancer (ToGA): a phase 3, open-label, randomised controlled trial. Lancet. (2010) 376:687–97. doi: 10.1016/S0140-6736(10)61121-X 20728210

[141] Bamias A Davis ID Galsky MD Arranz JÁ Kikuchi E Grande E. Atezolizumab monotherapy versus chemotherapy in untreated locally advanced or metastatic urothelial carcinoma (IMvigor130): final overall survival analysis from a randomised, controlled, phase 3 study. Lancet Oncol. (2024) 25:46–61. doi: 10.1016/S1470-2045(23)00539-9 38101431

[142] Hoffmann MJ Gaisa NT Nawroth R Ecke TH. Urothelial Carcinoma: Methods and Protocols. New York, NY, USA: Springer Nature (2023). doi: 10.1007/978-1-0716-3291-8

[143] Balar AV Kamat AM Kulkarni GS Uchio EM Boormans JL Roumiguié M. Pembrolizumab monotherapy for the treatment of high-risk non-muscle-invasive bladder cancer unresponsive to BCG (KEYNOTE-057): an open-label, single-arm, multicentre, phase 2 study. Lancet Oncol. (2021) 22:919–30. doi: 10.1016/S1470-2045(21)00147-9 34051177

[144] Cumberbatch MGK Jubber I Black PC Esperto F Figueroa JD Kamat AM. Epidemiology of bladder cancer: A systematic review and contemporary update of risk factors in 2018. Eur Urol. (2018) 74:784–95. doi: 10.1016/j.eururo.2018.09.001 30268659

[145] Bellmunt J de Wit R Vaughn DJ Fradet Y Lee J-L Fong L. Pembrolizumab as second-line therapy for advanced urothelial carcinoma. N Engl J Med. (2017) 376:1015–26. doi: 10.1056/NEJMoa1613683 PMC5635424 28212060

[146] Unger JM Vaidya R Albain KS LeBlanc M Minasian LM Gotay CC. Sex differences in risk of severe adverse events in patients receiving immunotherapy, targeted therapy, or chemotherapy in cancer clinical trials. J Clin Oncol. (2022) 40:1474–86. doi: 10.1200/JCO.21.02377 PMC9061143 35119908

[147] Berardi R Rossi F Papa R Appetecchia M Baggio G Bianchini M. Gender oncology: recommendations and consensus of the Italian Association of Medical Oncology (AIOM). ESMO Open. (2024) 9:102243. doi: 10.1016/j.esmoop.2024.102243 38394984 PMC10937209

[148] Zhang X Cheng L Gao C Chen J Liao S Zheng Y. Androgen signaling contributes to sex differences in cancer by inhibiting NF-κB activation in T cells and suppressing antitumor immunity. Cancer Res. (2023) 83:906–21. doi: 10.1158/0008-5472.CAN-22-2405 36634207

[149] Kwon H Schafer JM Song N-J Kaneko S Li A Xiao T. Androgen conspires with the CD8+ T cell exhaustion program and contributes to sex bias in cancer. Sci Immunol. (2022) 7:eabq2630. doi: 10.1126/sciimmunol.abq2630 35420889 PMC9374385

[150] Gandhi VD Cephus J-Y Norlander AE Chowdhury NU Zhang J Ceneviva ZJ. Androgen receptor signaling promotes Treg suppressive function during allergic airway inflammation. J Clin Invest. (2022) 132(4):e153397. doi: 10.1172/JCI153397 35025767 PMC8843736

[151] Walecki M Eisel F Klug J Baal N Paradowska-Dogan A Wahle E. Androgen receptor modulates Foxp3 expression in CD4+CD25+Foxp3+ regulatory T-cells. Mol Biol Cell. (2015) 26:2845–57. doi: 10.1091/mbc.E14-08-1323 PMC4571343 26063731

[152] Ejima A Abe S Shimba A Sato S Uehata T Tani-Ichi S. Androgens alleviate allergic airway inflammation by suppressing cytokine production in Th2 cells. J Immunol. (2022) 209:1083–94. doi: 10.4049/jimmunol.2200294 35977797

[153] Kissick HT Sanda MG Dunn LK Pellegrini KL On ST Noel JK. Androgens alter T-cell immunity by inhibiting T-helper 1 differentiation. Proc Natl Acad Sci U S A. (2014) 111:9887–92. doi: 10.1073/pnas.1402468111 PMC4103356 24958858

[154] Cheng MI Li JH Riggan L Chen B Tafti RY Chin S. The X-linked epigenetic regulator UTX controls NK cell-intrinsic sex differences. Nat Immunol. (2023) 24:780–91. doi: 10.1038/s41590-023-01463-8 PMC12882737 36928413

[155] Tang M Sun Y Huang C-P Chen L Liu B You B. High dose androgen suppresses natural killer cytotoxicity of castration-resistant prostate cancer cells via altering AR/circFKBP5/miRNA-513a-5p/PD-L1 signals. Cell Death Dis. (2022) 13:746. doi: 10.1038/s41419-022-04956-w 36038573 PMC9424293

[156] Liu Q You B Meng J Huang C-P Dong G Wang R. Targeting the androgen receptor to enhance NK cell killing efficacy in bladder cancer by modulating ADAR2/circ_0001005/PD-L1 signaling. Cancer Gene Ther. (2022) 29:1988–2000. doi: 10.1038/s41417-022-00506-w 35915245 PMC9750871

[157] Zhao R Chen X Ma W Zhang J Guo J Zhong X. A GPR174-CCL21 module imparts sexual dimorphism to humoral immunity. Nature. (2020) 577:416–20. doi: 10.1038/s41586-019-1873-0 31875850

[158] Aguilar-Pimentel JA Cho Y-L Gerlini R Calzada-Wack J Wimmer M Mayer-Kuckuk P. Increased estrogen to androgen ratio enhances immunoglobulin levels and impairs B cell function in male mice. Sci Rep. (2020) 10:18334. doi: 10.1038/s41598-020-75059-9 33110090 PMC7591566

[159] Altuwaijri S Chuang K-H Lai K-P Lai J-J Lin H-Y Young FM. Susceptibility to autoimmunity and B cell resistance to apoptosis in mice lacking androgen receptor in B cells. Mol Endocrinol. (2009) 23:444–53. doi: 10.1210/me.2008-0106 PMC2667704 19164450

[160] Ou Z Wang Y Liu L Li L Yeh S Qi L. Tumor microenvironment B cells increase bladder cancer metastasis via modulation of the IL-8/androgen receptor (AR)/MMPs signals. Oncotarget. (2015) 6:26065–78. doi: 10.18632/oncotarget.v6i28 PMC4694886 26305549

[161] Cioni B Zaalberg A van Beijnum JR Melis MHM van Burgsteden J Muraro MJ. Androgen receptor signalling in macrophages promotes TREM-1-mediated prostate cancer cell line migration and invasion. Nat Commun. (2020) 11:4498. doi: 10.1038/s41467-020-18313-y 32908142 PMC7481219

[162] Fang L-Y Izumi K Lai K-P Liang L Li L Miyamoto H. Infiltrating macrophages promote prostate tumorigenesis via modulating androgen receptor-mediated CCL4-STAT3 signaling. Cancer Res. (2013) 73:5633–46. doi: 10.1158/0008-5472.CAN-12-3228 PMC3833080 23878190

[163] Izumi K Fang L-Y Mizokami A Namiki M Li L Lin W-J. Targeting the androgen receptor with siRNA promotes prostate cancer metastasis through enhanced macrophage recruitment via CCL2/CCR2-induced STAT3 activation. EMBO Mol Med. (2013) 5:1383–401. doi: 10.1002/emmm.201202367 PMC3799493 23982944

[164] Huang C-K Pang H Wang L Niu Y Luo J Chang E. New therapy via targeting androgen receptor in monocytes/macrophages to battle atherosclerosis. Hypertension. (2014) 63:1345–53. doi: 10.1161/HYPERTENSIONAHA.113.02804 PMC4080890 24688120

[165] Lai J-J Lai K-P Chuang K-H Chang P Yu I-C Lin W-J. Monocyte/macrophage androgen receptor suppresses cutaneous wound healing in mice by enhancing local TNF-alpha expression. J Clin Invest. (2009) 119:3739–51. doi: 10.1172/JCI39335 PMC2786793 19907077

[166] Consiglio CR Gollnick SO. Androgen receptor signaling positively regulates monocytic development. Front Immunol. (2020) 11:519383. doi: 10.3389/fimmu.2020.519383 33193298 PMC7604537

[167] Becerra-Díaz M Strickland AB Keselman A Heller NM. Androgen and androgen receptor as enhancers of M2 macrophage polarization in allergic lung inflammation. J Immunol. (2018) 201:2923–33. doi: 10.4049/jimmunol.1800352 PMC6219904 30305328

[168] Xu P Yang JC Chen B Nip C Van Dyke JE Zhang X. Androgen receptor blockade resistance with enzalutamide in prostate cancer results in immunosuppressive alterations in the tumor immune microenvironment. J Immunother Cancer. (2023) 11:e006581. doi: 10.1136/jitc-2022-006581 37147019 PMC10163595

[169] Kohada Y Kaiho Y Takeda K Kuromoto A Ito J Teishima J. Analysis of the circulating myeloid-derived suppressor cells during androgen deprivation therapy for prostate cancer. IJU Case Rep. (2021) 4:367–70. doi: 10.1002/iju5.12351 PMC8560438 34755058

[170] Thompson MG Peiffer DS Larson M Navarro F Watkins SK. FOXO3, estrogen receptor alpha, and androgen receptor impact tumor growth rate and infiltration of dendritic cell subsets differentially between male and female mice. Cancer Immunol Immunother. (2017) 66:615–25. doi: 10.1007/s00262-017-1972-4 PMC11028910 28229217

[171] Consiglio CR Udartseva O Ramsey KD Bush C Gollnick SO. Enzalutamide, an androgen receptor antagonist, enhances myeloid cell-mediated immune suppression and tumor progression. Cancer Immunol Res. (2020) 8:1215–27. doi: 10.1158/2326-6066.CIR-19-0371 PMC7484281 32661092

[172] Hu C Pang B Lin G Zhen Y Yi H. Energy metabolism manipulates the fate and function of tumour myeloid-derived suppressor cells. Br J Cancer. (2020) 122:23–9. doi: 10.1038/s41416-019-0644-x PMC6964679 31819182

[173] Tang J-J Pan Y-F Chen C Cui X-L Yan Z-J Zhou D-X. Androgens drive sexual dimorphism in liver metastasis by promoting hepatic accumulation of neutrophils. Cell Rep. (2022) 39:110987. doi: 10.1016/j.celrep.2022.110987 35732131

[174] Markman JL Porritt RA Wakita D Lane ME Martinon D Noval Rivas M. Loss of testosterone impairs anti-tumor neutrophil function. Nat Commun. (2020) 11:1613. doi: 10.1038/s41467-020-15397-4 32235862 PMC7109066

[175] Scalerandi MV Peinetti N Leimgruber C Cuello Rubio MM Nicola JP Menezes GB. Inefficient N2-like neutrophils are promoted by androgens during infection. Front Immunol. (2018) 9:1980. doi: 10.3389/fimmu.2018.01980 30233581 PMC6129603

[176] Chuang K-H Altuwaijri S Li G Lai J-J Chu C-Y Lai K-P. Neutropenia with impaired host defense against microbial infection in mice lacking androgen receptor. J Exp Med. (2009) 206:1181–99. doi: 10.1084/jem.20082521 PMC2715023 19414555

[177] Ibáñez L Jaramillo AM Ferrer A de Zegher F. High neutrophil count in girls and women with hyperinsulinaemic hyperandrogenism: normalization with metformin and flutamide overcomes the aggravation by oral contraception. Hum Reprod. (2005) 20:2457–62. doi: 10.1093/humrep/dei072 15905296

[178] Alsamraae M Costanzo-Garvey D Teply BA Boyle S Sommerville G Herbert ZT. Androgen receptor inhibition suppresses anti-tumor neutrophil response against bone metastatic prostate cancer via regulation of TβRI expression. Cancer Lett. (2023) 579:216468. doi: 10.1016/j.canlet.2023.216468 37940068 PMC10710875

[179] Song W Li L He D Xie H Chen J Yeh C-R. Infiltrating neutrophils promote renal cell carcinoma (RCC) proliferation via modulating androgen receptor (AR) → c-Myc signals. Cancer Lett. (2015) 368:71–8. doi: 10.1016/j.canlet.2015.07.027 26231735

[180] Lin C Lin W Yeh S Li L Chang C. Infiltrating neutrophils increase bladder cancer cell invasion via modulation of androgen receptor (AR)/MMP13 signals. Oncotarget. (2015) 6:43081–9. doi: 10.18632/oncotarget.v6i40 PMC4767492 26517808

[181] Mazzeo L Ghosh S Di Cicco E Isma J Tavernari D Samarkina A. ANKRD1 is a mesenchymal-specific driver of cancer-associated fibroblast activation bridging androgen receptor loss to AP-1 activation. Nat Commun. (2024) 15:1038. doi: 10.1038/s41467-024-45308-w 38310103 PMC10838290

[182] Clocchiatti A Ghosh S Procopio M-G Mazzeo L Bordignon P Ostano P. Androgen receptor functions as transcriptional repressor of cancer-associated fibroblast activation. J Clin Invest. (2018) 128:5531–48. doi: 10.1172/JCI99159 PMC6264730 30395538

[183] Chen L Wang Y-Y Li D Wang C Wang S-Y Shao S-H. LMO2 upregulation due to AR deactivation in cancer-associated fibroblasts induces non-cell-autonomous growth of prostate cancer after androgen deprivation. Cancer Lett. (2021) 503:138–50. doi: 10.1016/j.canlet.2021.01.017 33503448

[184] Cioni B Nevedomskaya E Melis MHM van Burgsteden J Stelloo S Hodel E. Loss of androgen receptor signaling in prostate cancer-associated fibroblasts (CAFs) promotes CCL2- and CXCL8-mediated cancer cell migration. Mol Oncol. (2018) 12:1308–23. doi: 10.1002/1878-0261.12327 PMC6068356 29808619

[185] Liao C-P Chen L-Y Luethy A Kim Y Kani K MacLeod AR. Androgen receptor in cancer-associated fibroblasts influences stemness in cancer cells. Endocr Relat Cancer. (2017) 24:157–70. doi: 10.1530/ERC-16-0138 PMC5453797 28264911

[186] Leach DA Need EF Toivanen R Trotta AP Palethorpe HM Tamblyn DJ. Stromal androgen receptor regulates the composition of the microenvironment to influence prostate cancer outcome. Oncotarget. (2015) 6:16135–50. doi: 10.18632/oncotarget.v6i18 PMC4599261 25965833

[187] Torres-Estay V Carreño DV San Francisco IF Sotomayor P Godoy AS Smith GJ. Androgen receptor in human endothelial cells. J Endocrinol. (2015) 224:R131–7. doi: 10.1530/JOE-14-0611 PMC4700832 25563353

[188] Guan Z Li C Fan J He D Li L. Androgen receptor (AR) signaling promotes RCC progression via increased endothelial cell proliferation and recruitment by modulating AKT → NF-κB → CXCL5 signaling. Sci Rep. (2016) 6:37085. doi: 10.1038/srep37085 27848972 PMC5111066

[189] Eisermann K Broderick CJ Bazarov A Moazam MM Fraizer GC. Androgen up-regulates vascular endothelial growth factor expression in prostate cancer cells via an Sp1 binding site. Mol Cancer. (2013) 12:7. doi: 10.1186/1476-4598-12-7 23369005 PMC3616929

[190] Boddy JL Fox SB Han C Campo L Turley H Kanga S. The androgen receptor is significantly associated with vascular endothelial growth factor and hypoxia sensing via hypoxia-inducible factors HIF-1a, HIF-2a, and the prolyl hydroxylases in human prostate cancer. Clin Cancer Res. (2005) 11:7658–63. doi: 10.1158/1078-0432.CCR-05-0460 16278385

[191] Godoy A Watts A Sotomayor P Montecinos VP Huss WJ Onate SA. Androgen receptor is causally involved in the homeostasis of the human prostate endothelial cell. Endocrinology. (2008) 149:2959–69. doi: 10.1210/en.2007-1078 PMC2408806 18292195

[192] Yoshida S Aihara K-I Ikeda Y Sumitomo-Ueda Y Uemoto R Ishikawa K. Androgen receptor promotes sex-independent angiogenesis in response to ischemia and is required for activation of vascular endothelial growth factor receptor signaling. Circulation. (2013) 128:60–71. doi: 10.1161/CIRCULATIONAHA.113.001533 23723256 PMC3933182

[193] Yu J Akishita M Eto M Ogawa S Son B-K Kato S. Androgen receptor-dependent activation of endothelial nitric oxide synthase in vascular endothelial cells: role of phosphatidylinositol 3-kinase/akt pathway. Endocrinology. (2010) 151:1822–8. doi: 10.1210/en.2009-1048 20194727

[194] Powles T Yuen KC Gillessen S Kadel EE 3rd Rathkopf D Matsubara N. Atezolizumab with enzalutamide versus enzalutamide alone in metastatic castration-resistant prostate cancer: a randomized phase 3 trial. Nat Med. (2022) 28:144–53. doi: 10.1038/s41591-021-01600-6 PMC9406237 35013615

[195] Pu Y Xu M Liang Y Yang K Guo Y Yang X. Androgen receptor antagonists compromise T cell response against prostate cancer leading to early tumor relapse. Sci Transl Med. (2016) 8(333):333ra47. doi: 10.1126/scitranslmed.aad5659 27053771

[196] Arora VK Schenkein E Murali R Subudhi SK Wongvipat J Balbas MD. Glucocorticoid receptor confers resistance to antiandrogens by bypassing androgen receptor blockade. Cell. (2013) 155:1309–22. doi: 10.1016/j.cell.2013.11.012 PMC3932525 24315100

[197] Conforti F Pala L Pagan E Bagnardi V De Pas T Queirolo P. Sex-based dimorphism of anticancer immune response and molecular mechanisms of immune evasion. Clin Cancer Res. (2021) 27:4311–24. doi: 10.1158/1078-0432.CCR-21-0136 PMC7611463 34016641

[198] Cha H-R Lee JH Ponnazhagan S. Revisiting immunotherapy: A focus on prostate cancer. Cancer Res. (2020) 80:1615–23. doi: 10.1158/0008-5472.CAN-19-2948 PMC7641094 32066566

[199] Rebuzzi SE Rescigno P Catalano F Mollica V Vogl UM Marandino L. Immune checkpoint inhibitors in advanced prostate cancer: current data and future perspectives. Cancers. (2022) 14(5):1245. doi: 10.3390/cancers14051245 35267553 PMC8909751

[200] Kim S-E Paik HY Yoon H Lee JE Kim N Sung M-K. Sex- and gender-specific disparities in colorectal cancer risk. World J Gastroenterol. (2015) 21:5167–75. doi: 10.3748/wjg.v21.i17.5167 PMC4419057 25954090

